# Biopolymer-Based Sustainable Food Packaging Materials: Challenges, Solutions, and Applications

**DOI:** 10.3390/foods12122422

**Published:** 2023-06-20

**Authors:** Kalpani Y. Perera, Amit K. Jaiswal, Swarna Jaiswal

**Affiliations:** 1 Sustainable Packaging and Bioproducts Research (SPBR) Group, School of Food Science and Environmental Health, Faculty of Sciences and Health, Technological University Dublin, City Campus, Grangegorman, D07 ADY7 Dublin, Ireland; kalpani.gamage@tudublin.ie (K.Y.P.); swarna.jaiswal@tudublin.ie (S.J.); 2 Environmental Sustainability and Health Institute, Technological University Dublin, City Campus, Grangegorman, D07 H6K8 Dublin, Ireland

**Keywords:** biopolymers, food packaging materials, bioplastics, sustainability, biopolymer-based materials, SWOT analysis

## Abstract

Biopolymer-based packaging materials have become of greater interest to the world due to their biodegradability, renewability, and biocompatibility. In recent years, numerous biopolymers—such as starch, chitosan, carrageenan, polylactic acid, etc.—have been investigated for their potential application in food packaging. Reinforcement agents such as nanofillers and active agents improve the properties of the biopolymers, making them suitable for active and intelligent packaging. Some of the packaging materials, e.g., cellulose, starch, polylactic acid, and polybutylene adipate terephthalate, are currently used in the packaging industry. The trend of using biopolymers in the packaging industry has increased immensely; therefore, many legislations have been approved by various organizations. This review article describes various challenges and possible solutions associated with food packaging materials. It covers a wide range of biopolymers used in food packaging and the limitations of using them in their pure form. Finally, a SWOT analysis is presented for biopolymers, and the future trends are discussed. Biopolymers are eco-friendly, biodegradable, nontoxic, renewable, and biocompatible alternatives to synthetic packaging materials. Research shows that biopolymer-based packaging materials are of great essence in combined form, and further studies are needed for them to be used as an alternative packaging material.

## 1. Introduction

The use of biopolymers as packaging materials is becoming an emerging trend worldwide due to their major benefits over plastics, such as biodegradability, eco-friendly nature, nontoxicity, and biocompatibility. These natural biopolymers have excellent film-forming cohesive structures and thin protective layers of film [1].

**Figure 1:**
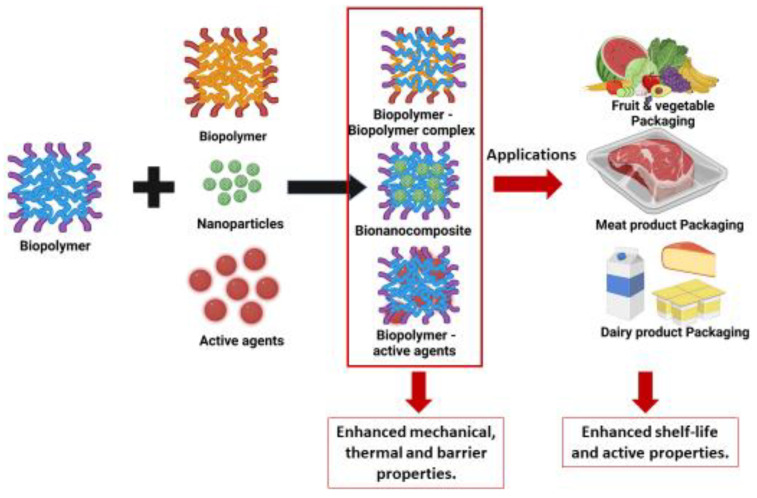
An overview of biopolymers in food packaging (Figure created with BioRender).

Biopolymers used as food packaging materials are mainly polysaccharides, proteins, and aliphatic polyesters, which can maintain food quality and increase the shelf-life of the product. These packaging materials [1] have barrier properties that control the exchange of gases, moisture, aroma, and lipids from the external environment and vice versa, [2] possess antimicrobial activity that can protect the food product from the external environment, and [3] prevent the loss of desirable compounds such as flavor and texture [2,3,4]. Biopolymers such as starch, cellulose, and polylactic acid (PLA) are currently used for food packaging materials. However, the main limitation of using biopolymers in food packaging is their weak mechanical strength and high sensitivity to moisture. The merits and demerits differ depending on the type of biopolymer used for food packaging. Table 1 shows the advantages and disadvantages of different biopolymers used in food packaging. To overcome the weaknesses of biopolymers, many studies have been performed with the addition of reinforcing agents such as nanofillers, biopolymers, plasticizers, and natural agents such as essential oils. Furthermore, biopolymer matrices act as carriers for antimicrobial substances, antioxidants, flavor agents, vitamins, or nutrients, thereby aiding in improving food quality, safety, nutritional value, and sensory properties. An overview of biopolymers in food packaging is presented in Figure 1. Due to the numerous advantages, biopolymers have been proposed as an alternative to synthetic polymers such as plastic, which reduces the harmful impact on the environment [5]. As the use of biopolymers in food packaging materials is increasing, it is important to upgrade the biopolymer industry to a large scale. Strategies for sourcing biopolymers include utilizing agricultural waste, instituting efficient cultivation practices, and researching innovative biopolymer production technologies.

Despite numerous works reporting on the use of biomaterials for packaging [61,71,72], the novelty of this review lies in the extensive explanation of each material’s unique qualities that make it appealing for packaging a specific food product. This review article also describes various challenges and possible solutions associated with food packaging materials. It examines the current state of research and industrial application, including the advantages and disadvantages of various biopolymer-based food packaging. Finally, a SWOT (strengths, weaknesses, opportunities, and threats) analysis is performed on biopolymers, and future trends are discussed. Thus, this review will be useful in the decision-making process to develop biopolymer-based packaging materials.

## 2. Current Food Packaging Materials and Associated Issues/Challenges

Plastic, a petroleum-based, diverse, and ubiquitous material, is widely used in food packaging due to its lightweight, cost-effective, transparent, versatile, and easy-to-process properties [1,5]. These synthetic polymers possess excellent mechanical, thermal, and barrier characteristics [1,5], while ultra-thin layers extend the shelf-life of packaged products and reduce food waste [5]. Consequently, plastics provide direct economic benefits by lowering transportation costs.

Global plastic production has increased significantly, with 40% of all produced plastic being used for packaging, and nearly half of that for food packaging [47,73,74]. Europe’s plastic distribution demand is dominated by packaging at 39.6% [75]. However, plastic’s high production volume, short usage time, non-biodegradable nature, and inadequate management have raised concerns worldwide, with recycling challenges arising from multilayer plastics [5,47,76].

Plastics account for about 6% of global oil consumption, projected to increase to 20% by 2050 [5]. Plastic waste damages terrestrial environments and pollutes aquatic ones, accumulating due to prolonged degradation. Landfill plastics release harmful substances during abiotic and biotic degradation, contaminating soil and water [77]. Chlorinated plastics leach toxic chemicals, polluting ecosystems, while plastic degradation in water releases chemicals such as polystyrene and Bisphenol A, causing water pollution [77]. Methane and CO_2_ emissions during plastic microbial digestion contribute to global warming [77]. Animals are exposed to plastic waste through ingestion and entanglement, with detrimental consequences.

Countries are addressing plastic pollution through waste reduction, production reduction, recycling, and alternatives [77]. Governments have adopted policy initiatives to reduce plastic pollution, with global legislation focusing on protecting territorial and marine environments. The United Nations Convention on the Law of the Sea (UNCLOS) in 1982 was the first international legislation agreement on plastic waste [77]. Other conventions include the International Convention for the Prevention of Pollution from Ships (MARPOL) in 1973, the London Convention (1972), the Global Program Action for the Protection of the marine environment from land-based activities (GPA) in 1995, and the Global Partnership on Marine Litter (GPML) formed in 2012 [77].

The 17 Sustainable Development Goals (SDGs) by the United Nations General Assembly in 2015 aim to promote sustainability, protect ecological life support systems, and reduce waste and pollution by 2030 [77]. The Basel Convention (1989), Rotterdam Convention (2004), and Stockholm Convention (2004) address the safe disposal and management of hazardous substances associated with plastic disposal [77]. Legislation on global warming includes the United Nations Framework Convention on Climate Change (1992) (UNFCCC) and the Montreal Protocol (1987) [77].

The European Union (EU) combats plastic pollution through strategic legislation, including the EU action plan in 2015, the Regional Strategy for Plastics in a Circular Economy in 2018, and the directive on the reduction in the impact of certain plastic products in 2019 [78,79]. The latest update in 2020 focuses on the regulation of recycled content, waste reduction, and product labeling [75].

## 3. Possible Solutions for Current Food Packaging Materials

The growing environmental concerns surrounding plastics have prompted research into alternative food packaging materials [79]. Biodegradable materials, such as biopolymers, bioplastics, bio-nanocomposites, and edible coatings, are being developed to replace plastics.

Biodegradable polymers are renewable, nontoxic, biodegradable, biocompatible, reproducible, versatile, abundantly available, and boast a low carbon footprint [3,47]. However, issues such as viscosity, hydrophobicity, crystallization activity, brittleness, water sensitivity, thermal stability, gas barrier properties, mechanical strength, processing difficulty, and cost have hindered their widespread industrial adoption [2]. Biodegradable polymers can be classified as polysaccharides (starch, cellulose, chitosan, etc.), proteins (soy protein, collagen, zein, etc.), and aliphatic polyesters (polybutylene adipate terephthalate (PBAT), PLA, etc.) [1].

To address these issues, biodegradable polymers can be blended with other biodegradable polymers, plasticizers (e.g., glycerol), and compatibilizers (e.g., essential oils) [2,3,80]. The biopolymer packaging market in Europe increased from 1743.9 million m^2^ in 2016 to 2427.1 million m^2^ in 2021 [78]. Bioplastics are bio-based and/or biodegradable plastics that share properties with traditional plastics and offer additional benefits such as renewability and biodegradability [81].

Bio-nanocomposites, which consist of a bio-based polymer matrix and an organic/inorganic filler with at least one nanoscale material, are suitable as active and/or intelligent packaging materials due to their enhanced mechanical, thermal, barrier, antimicrobial, and antioxidant properties [82,83,84]. These materials focus on extending shelf-life and reducing microbial growth in food products [83,84].

Biopolymer-based edible films, formed from polysaccharides or blends of polysaccharides containing proteins, lipids, and food-grade additives, are suitable for human consumption and can increase the shelf-life and quality of food products [85,86]. Despite their potential, these packaging techniques confront obstacles such as poor elongation, safety and health concerns, high cost, processing difficulties, lack of awareness, cultural concerns, and customer acceptance [87].

## 4. Degradation Chemistry of Biopolymers

During biopolymer biodegradation, the polymers are first converted to monomers, and they are then mineralized. The mineralization of the organic material takes place by microorganisms (e.g., fungi, archaea, and bacteria) eventually resulting in carbon dioxide, water, and biomass. The reactions occurring during biopolymer biodegradation are as below:

Biodegradable polymers → CO_2_+ H_2_O + biomass

The biodegradation of the large molecules of the biopolymers takes place by extracellular enzymes in microorganisms, while the smaller molecules are transported into the microorganism digestion by endoenzymes. For the biodegradation of a biopolymer substrate, most microorganisms use multiple enzyme systems. The biopolymer biodegradation takes place either through oxo-biodegradation or hydro-biodegradation. Oxo-biodegradation takes place in natural polymers such as rubber, humus, and lignin. During this process, loss of the mechanical properties of carbohydrate polymers takes place by the peroxidation process, which is initiated by heat/light, resulting in oxocarboxylic acid molecules, aldehydes, ketones, and alcohols. After that, the biopolymers undergo bio-assimilation with the aid of enzymes of microorganisms into the water, carbon dioxide, and biomass. The hydro-biodegradation process takes place in cellulose, starch, and aliphatic polyesters. The biopolymers are converted into monomers through the enzymatic digesting of microorganisms. The hydrolysis of ester bonds in monomers is performed by the extracellular enzymes of microorganisms. The aliphatic polyesters and carbohydrate polymers are hydrolyzed and bio-assimilated rapidly in an aqueous medium [88].

The rate of biodegradation depends upon different factors such as (1) polymer characteristics (chemical bonds, branching, hydrophilicity/hydrophobicity, stereochemistry, molecular weight, chain flexibility, crystallinity, interactions with polymers, coatings, surface area, mobility, and addition of plasticizers/additives/active agents), (2) microorganism type (aerobic and anaerobic facultative, co-metabolism, nature, enzymes, enzyme level, enzyme location, enzyme kinematics, and inhibitors/ inducers), and (3) environment conditions (temperature, humidity, oxygen, salts, metals, trace nutrients, pH, redox potential, stability, pressure, alternate carbon, and light). When the above conditions are present appropriately, the rapid degradation process occurs. During industrial composting, the bioplastics are biodegraded in approximately 6–12 weeks [89].

To access the biodegradability of a biopolymer, laboratory tests, simulation tests, and field tests are carried out. The laboratory tests applied include enzyme tests, clear zone tests, Sturm tests, and synthetic environment-defined conditions. Stimulation tests are performed using laboratory reactors, water, soil, compost, and material from landfills with complex environments in defining conditions. Finally, field tests are performed in nature, water, and soil/compland fill under a complex environment in variable conditions [90].

## 5. Important Properties of Biopolymers in Food Packaging

The properties of the packaging materials, such as barrier, mechanical, chemical, and thermal properties, play an important role in increasing the shelf-life and maintaining the quality of the food products. The barrier properties of a biopolymer used in food packaging are the main parameter for extending the shelf-life of the packed food product. Barrier properties such as gas, water vapor, organic vapors, and liquids are essential for food packaging to separate the food product from the external environment. In addition, these products differ in the different biopolymers used in food packaging. Thus, the loss/gain of oxygen and water plays a major role in food deterioration. The barrier properties play a crucial role in packaging since gas/water vapor may pass through the walls of the biopolymer, resulting in changing the food product quality and shelf-life [91,92].

The gas permeability of the packaging material depends on the parameters; transmission rate, permeance, and permeability. However, the barrier properties of materials not only depend on these factors but also on environmental conditions such as temperature, pressure, and relative humidity. Further, the rating of the barrier properties also depends on the nature of the food products that are to be packaged. As a result, food packaging materials can prolong the shelf-life of food products by improving the barrier properties [93].

The oxygen barrier properties of a packaging material play an important role in the preservation of fresh food products. The oxygen permeability is quantified by the oxygen transmission rate and oxygen permeability [93]. This measures the amount of oxygen in the packaging system. When the oxygen permeability is reduced, the oxygen pressure in the packaging system drops, resulting in an extended shelf-life of the food product [93].

The water vapor barrier properties are of great significance for food products to maintain physical or chemical deterioration concerning the moisture content. The water vapor barrier properties are quantified by the water vapor permeability of the packaging material by the ASTM E-96-95 standard method and the water vapor transmission rate [94]. The water vapor permeability depends upon the solubility and the diffusion of the water in the polymer material. The shelf-life of some food products is directly related to the water exchange rate between the external and internal environment; thus, the water transfer should be reduced to protect the food product from moisture [95].

The UV barrier properties of packaging material are quantified by the optical properties of a film using a spectrophotometer [96]. The UV barrier properties are essential to prevent the loss of nutrient value and the change in the color of food [29].

Mechanical properties of the packaging system are essential to secure the food during stressful conditions such as storage, handling, and processing of the food. The architecture of the polymer matrix is the key factor that determines the mechanical properties of the biopolymer. The mechanical properties of packaging material are determined by tensile properties such as tensile strength, elongation at break, and elastic modulus [30,34,97].

Chemical resistance is important because the food in the package may be acidic and combine with the packaging material. For safety reasons, it is important to find out what the food is made of chemically before packing it. When these chemicals combine with and become absorbed by the biopolymer matrix, the mechanical properties of the material may change [92].

The thermal properties of the packaging material are determined by thermogravimetric and differential scanning calorimetry. Thermal properties and thermal stability are essential for the heat resistance of the packaging material. Thus, the thermal properties allow us to store and transport the food packaging at the temperature essential for the food products [57].

## 6. Biodegradable Polymers Currently Used in the Food Packaging Industry

### 6.1. Polysaccharide-Based Biopolymers

Polysaccharide-based biopolymers are nontoxic, abundantly available natural components that are highly suitable as food packaging materials. They have excellent mechanical and structural properties while being selectively permeable to carbon dioxide and oxygen. However, they have poor water vapor barrier properties [66,97,98].

The biodegradable films from biodegradable polymers have been modified by the addition of various reinforcement agents to produce a packaging system that has beneficial properties and is suitable for industrial applications. Such films may include polymer blending/hybrid films, plasticizers, and/or nanoparticles (NPs). The addition of antioxidants, antimicrobials, nutrients, and color change indicators such as essential oils, phenolic compounds, and plant extracts to biopolymers make them attractive, active, and intelligent packaging, increasing the shelf-life of food. The addition of plasticizers (such as glycerol/sorbitol) to biopolymers can modify their brittleness, increase their processing ability, increase the mobility of starch chains, and decrease moisture absorption. Unfortunately, the incorporation of plasticizers into biodegradable polymers decreases their mechanical properties [6,23].

**Figure 2:**
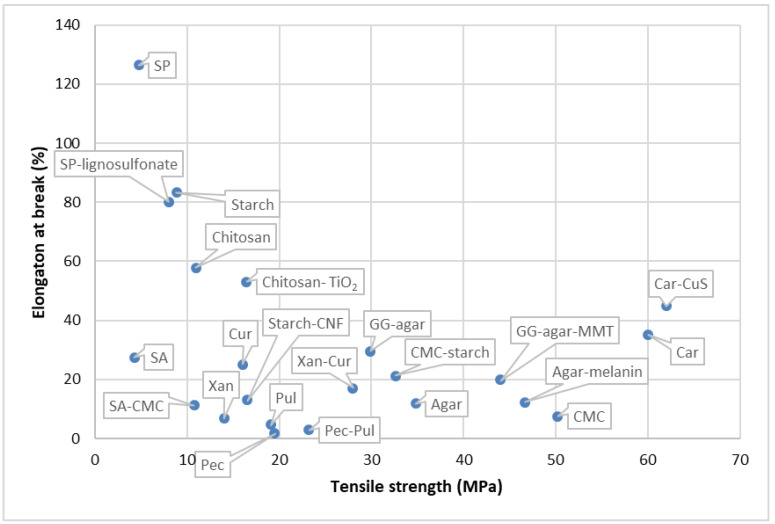
Elongation at break versus tensile strength for polysaccharide-based biopolymers. Abbreviations: CNF-cellulose nanofibers, GG—Gellan gum, MMT—montmorillonite, SA—sodium alginate, CMC—carboxymethyl cellulose, Pec—pectin, Pul—pullulan, Car—carrageenan, SP—soy protein, Cur—Curdlan, Xan—xanthan.

To improve the properties of the polysaccharides and develop them into an industrially used active/smart packaging system, a combination of polysaccharides, lipids, and NPs are utilized as explained in detail below in the different sub-sections of polysaccharides (Table 2). The mechanical properties of some of the studies depicted in Table 2 have been taken, and a scatter plot has been drawn for a better understanding of the properties, which can be seen in Figure 2. The active polysaccharides can be extracted using different methods, such as hot water extraction, acid–base extraction, enzyme extraction, ultrasonic extraction, ultrahigh pressure extraction, microwave extraction, and supercritical fluid extraction [97]. Currently, starch blends and cellulose are used in industrial applications. Products such as cups, plates, cutlery, and food packs are produced from thermoplastic starch. Companies such as Plantic Technologies (Plantic, USA), Rodenburg Biopolymers (Solanyl, Tokyo, Japan), Biotec (Bioplast, Bristol, PA, USA), Biop (Biopar, Schwalbach am Taunus, Germany), Novamont (Mater Bi, Bottrighe, Italy/Waltham, MA, USA), KINGFA (ECOPOND^®^, Guangzhou, China), and Biome Bioplastics Limited (BIOME Bioplastics, Southampton, UK) manufacture starch-based films [81,99]. Additionally, cellulose is used in industries to make bags, wraps for food, and films. Companies such as Nature Works LLC (BioMass Packaging^®^, Richmond, CA, USA) and Nature Flex™ (Innovia Films, Wigton, UK) produce these cellulose biopolymers on a large scale. Nature Flex™ manufactures cellulose-based products such as coffee and tea packaging, compostable snack bags, packaging for dried foods, compostable stick packs, packaging for chocolate and candy, compostable packaging, packaging for bakeries, custom packaging for food service, and compostable bags. In addition, cellulose and chitosan-lined paper bags and cups are also produced at the industrial level.

#### 6.1.1. Starch

Starch is a polysaccharide composed of linear (amylose) and branched (amylopectin) sections, which are extracted from maize, potato, cassava, and cereal grains. It is regarded as one of the most promising biodegradable polymers for use in food packaging due to its many advantageous properties, such as biodegradability, low cost, abundance, transparency, colorlessness, flavorlessness, tastelessness, reduced water sensitivity, excellent oxygen barrier properties, renewability, edibility, and being an excellent film-forming biopolymer. Nevertheless, starch alone is not a suitable food packaging material as it lacks basic important properties such as vapor barrier, mechanical, and thermal properties. It is also found to be brittle due to the massive inter- and intra-molecular interactions between starch chains and is hydrophilic [4,6,7,30,100,101,102,103]. The two main techniques, the dry process, and the wet process, are used for the development of starch biofilms [104].

Some limitations of using starch as a packaging material can be overcome by chemical or physical modification of native starch. Chemical modification has limitations since it is a complex, time-consuming process that can be toxic. The physical modification of starch such as microwave treatment, pulsed electric field processing, and high-pressure and irradiation treatments has yielded promising results [105]. Thermoplastic starch (TPS) is developed by transforming the starch into a melted material by adding a plasticizer. However, TPS also has poor mechanical and water vapor barrier properties, which make it unsuitable for food packaging without the addition of other components [8,106]. Another modification of starch is the development of starch-based aerogels, which have been highly studied and are still in the basic research stage. These aerogels are environmentally friendly, biodegradable, and have many unique properties. The two main fabrication routes of starch-based aerogels based on their shapes are monolith and microsphere [101]. Moreover, starch nanocrystals are produced from native starch, which has many merits, such as high surface area, robust mechanical properties, and intriguing self-assembly properties [107]. Starch foam packaging is an alternative packaging that was developed for polystyrene foam in the initial research phase [103].

**Figure 3:**
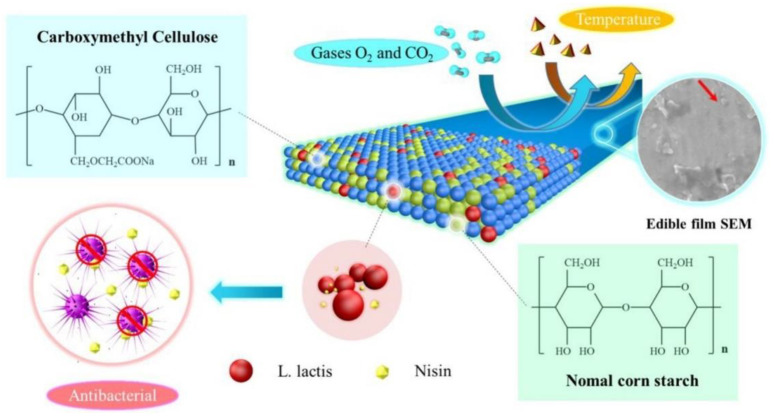
Antimicrobial edible film encapsulating *Lactococcus lactis* into a starch-carboxymethyl cellulose matrix. Reprinted/adapted with permission from Ref. [108]. 2021, Elsevier.

The incorporation of starch with other biodegradable polymers or synthetic polymers allows for better packaging material. Recent studies by Lan et al. [108] focused on encapsulating *Lactococcus lactis* into a starch-carboxymethyl cellulose matrix to form an antimicrobial edible film as shown in Figure 3 with low moisture content. The film containing 1.5% *L. lactis* had the lowest water vapor transmission rate (5.54 g/m s Pa) and retained a viable count of 5.64 log CFU/g of *L. lactis* after 30 days. After 8 days, the film containing 1.5% *L. lactis* had the maximum nisin release (3.35 mg/mL) and antibacterial efficacy against *Staphylococcus aureus* (53.53%).

**Table 2:** Applications of polysaccharide-based biopolymers in food packaging.

Packaging Material	Characteristics of Food Packaging System	Mechanical Properties	Thermal Properties	Application	Reference
**Starch**					
Starch-cinnamon essential oil (CEO)—TiO_2_ NPs	Improved oxygen, mechanical properties, and water vapor permeability Decreased barrier properties Antimicrobial activity against *E. coli*, *S. typhimurium*, and *S. aureus*	TS (MPa): ~18, ~25(respectively for starch, starch—5% TiO_2_—3% CEO) EB (%): ~26, ~24 (respectively, for starch, starch—5% TiO_2_—3% CEO)	-	Potential active food packaging material for fresh pistachio packaging	[6]
Starch—PBAT	Improved flexibility, water vapor barrier, mechanical properties, and hydrophobicity	TS (MPa): 1.5, 7.4 MPa (respectively, for starch—0% PBAT, starch—50 wt% PBAT) EB (%): ~100, ~450 (respectively, for starch—0% PBAT, starch—50 wt% PBAT)	-	Potential active food packaging material	[9]
Starch—cellulose nanofibers (CNF)	Improved water barrier, optical and mechanical	TS (MPa): 8.9 ± 0.1, 16.5 ± 0.4 (respectively, for starch, starch—10% CNF) EB (%): 83.2 ± 0.7, 13.2 ± 1.2(respectively, for starch, starch—10% CNF	-	Potential active food packaging material	[105]
Starch—cellulose nanocrystals (CNC)	Improved tensile strength, Young’s modulus, and mechanical properties. Decreased water vapor permeability	TS (MPa): ~16, ~24 (respectively, for starch, starch—15% CNC) EB (%): ~13, ~4 (respectively, for starch, starch—15% CNC)	T_onset_ (°C): 307 ± 3.21, 266 ± 6.03 (respectively, for starch, starch—15% CNC) Tmax (°C): 335 ± 2.65, 328 ± 1.53 (respectively, for starch, starch—15% CNC)	Potential active food packaging material	[7]
Starch—ZnO-rosemary polyphenols included in PVA	Improved Young’s modulus, stress and strain at break, and tensile toughness Decreased water vapor permeability Antimicrobial activity against *E. coli* and antioxidant activity	TS (MPa): 2.1 ± 0.2, 3.5 ± 0.2 (respectively, for starch, starch—ZnO-rosemary polyphenols included in PVA) EB (%): 50 ± 16, 76 ± 9 (respectively, for starch, starch–ZnO-rosemary polyphenols included in PVA)	-	Potential active food packaging material	[8]
Starch—cellulose nanofiber (CNF)-thymol	Improved the water vapor barrier Tensile strength and Young’s modulus decreased, and the elongation at break increased with increasing thymol concentration Antibacterial activity against *E. coli*	TS (MPa): ~11, ~6 (respectively, for starch—CNF, starch—CNF-10% thymol) EB (%): ~110, ~122 (respectively, for starch—CNF, starch—CNF-10% thymol)	-	Potential active food packaging material	[106]
Cassava starch—red cabbage extract	Colorimetric reaction to pH change (2–12) and volatile ammonia visible with the naked eye	TS (MPa): 5.73 ± 0.12, 10.37 ± 0.22 (respectively, for native cassava starch, oxidized-acetylated starch) EB (%): 102.44 ± 3.2, 60.52 ± 3.39 (respectively, for native cassava starch, oxidized-acetylated starch)	Weight losses of the first phase (30–105 °C), the second phase (106–320 °C), and the third phase (above 320 °C)	Potential intelligent food packaging material.	[107]
Chitosan					
Chitosan—polyvinyl alcohol-anthocyanins	Improved tensile strength, hydrophobic and barrier properties	-	-	Potential intelligent food packaging material for real-time shrimp freshness monitoring	[109]
Chitosan—cellulose acetate phthalate—ZnO NPs	Improved thermal stability and barrier properties Reduced water contact angle. Biodegradation of 30 to 50% of film in 28 days	TS (MPa): 8.29 ± 0.16, 9.83 ± 0.19 (respectively, for chitosan, chitosan—cellulose acetate phthalate—ZnO NPs) EB (%): 12.67 ± 0.38, 15.44 ± 0.46 (respectively, for chitosan, chitosan—cellulose acetate phthalate—ZnO NPs)	-	Potential active food packaging material for black grapefruits by increasing shelf-life up to 9 days	[110]
Chitosan—TiO_2_ NPs	Exhibit photodegradation activity when exposed to UV light, thus delaying ripening process and changes in quality of tomatoes	TS (MPa): 10.96 ± 1.57, 16.43 ± 0.46 (respectively, for chitosan, chitosan—TiO_2_ NPs) EB (%): 57.71 ± 1.28 53.06 ± 2.15 (respectively, for chitosan, chitosan—TiO_2_ NPs)	-	Potential active food packaging material to maintain quality and extend storage life of climacteric fruit	[111]
Chitosan—TiO_2_ NPs—*Cymbopogon citratus* essential oil	Incorporation of TiO_2_ NPs increased water vapor permeability and tensile strength. Decreased elongation at break and film solubility	TS (MPa): 37.50 ± 0.00, 62.46 ± 0.13 (respectively, for chitosan, chitosan—1%TiO_2_ NPs—0.5% Cymbopogon citratus essential oil) EB (%): 4.77 ± 0.03, 4.81 ± 0.01 (respectively, for chitosan, chitosan—1%TiO_2_ NPs—0.5% Cymbopogon citratus essential oil)	-	Potential active packaging material for prolong shelf-life of minced meat by reducing microbial growth	[112]
Chitosan—graphene oxide NPs	Improved tensile strength, Young’s modulus, and antimicrobial activity Decreased water vapor permeability	TS (MPa): 0.063 ± 0.0041, 0.083 ± 0.0034 (respectively, for chitosan, chitosan—graphene oxide NPs) EB (%): 6.45 ± 0.05, 6.95 ± 0.72 (respectively, for chitosan, chitosan—graphene oxide NPs)	-	Potential active food packaging bag for prolonging shelf-life of melon fruits	[12]
Chitosan-pullulan—clove-essential-oil-loaded chitosan–ZnO hybrid NPs	Increased tensile strength, hydrophobicity, UV light blocking ability, water vapor barrier and oxygen barrier properties Enhanced antioxidant activity Antibacterial activity against *P. aeruginosa*, *S. aureus*, and *E. coli* Extend the shelf-life of chicken meat by up to 5 days	TS (MPa): 62.0 ± 0.2, 83.7 ± 0.2 (respectively, for chitosan, chitosan-pullulan—clove-essential-oil-loaded chitosan—3% ZnO hybrid NPs) EB (%): 5.0 ± 0.1, 5.1 ± 0.5 (respectively, for chitosan, chitosan-pullulan—clove-essential-oil-loaded chitosan—3% ZnO hybrid NPs)	-	Potential active packaging material for prolonging shelf-life of chicken	[113]
Chitosan—modified silica NPs	Improved mechanical, water vapor barrier, and UV light barrier properties Increased antioxidant activity	TS (MPa): 101.29 ± 0.57, 125.25 ± 0.36 (respectively, for chitosan, chitosan—4% modified silica NPs) EB (%): 4.78 ± 0.06, 2.26 ± 0.11 (respectively, for chitosan, chitosan—4% modified silica NPs		Potential antioxidant active packaging material	[114]
Chitosan-alginate—TiO_2_ NPs	Improved mechanical, UV barrier, antibacterial, and biodegradability properties antimicrobial activity against foodborne pathogens *E. coli*, *S. aureus*, *S. typhi*, and *L. monocytogene*	TS (MPa): 1.82 ± 0.16, 26.86 ± 0.28 (respectively, for chitosan—alginate, chitosan-alginate—0.2% TiO_2_ NPs) EB (%): 2.05 ± 0.64, 3.66 ± 0.63 (respectively, for chitosan –alginate, chitosan-alginate—0.2% TiO_2_ NPs)	The first stage of weight loss is the temperature range of 60–180 °C The second stage of weight loss takes place in the temperature range of 210–400 °C	Potential active food packaging bag for prolonging shelf-life of cherry tomatoes	[29]
Carrageenan					
Carrageenan—CuS NPs	Improved mechanical and thermal properties Antimicrobial activity against *E. coli* and *S. aureus*	TS (MPa): ~60, ~62 (respectively, for carrageenan, carrageenan—0.15% CuS NPs EB (%): ~35, ~45(respectively, for carrageenan, carrageenan—0.15% CuS NPs	First step occurred at about 60–110 °C The second stage from 120 to 220 °C The third stage, from 230 to 290 °C	Potential antimicrobial active food packaging material for beef packaging	[115]
κ-carrageenan—Zataria multiflora extract—nanoclay	Improved UV barrier, mechanical and thermal properties Antimicrobial activity against *E. coli* and *P. aeruginosa*	TS (MPa): 17.0 ± 2.0, 33.7 ± 3.9 (respectively, for κ-carrageenan-nanoclay, κ-carrageenan-Zataria multiflora extract—nanoclay) EB (%): 63.8 ± 16.8, 20.9 ± 5.7 (respectively, for κ-carrageenan-nanoclay, κ-carrageenan- Zataria multiflora extract—nanoclay)	Initial step of weight loss around 40–120 °C The second step around 120–260 °C The third weight loss stage from 260 to 500 °C	Potential active food packaging material	[16]
κ-carrageenan—pomegranate flesh and peel extracts	Improved tensile strength, water vapor, and UV light barrier properties High antioxidant, antimicrobial, and pH-sensitive properties	TS (MPa): 24.73 ± 1.25, 30.94 ± 0.85 (respectively, for κ-carrageenan, κ-carrageenan—pomegranate flesh and peel extracts) EB (%): 13.82 ± 2.45, 22.29 ± 1.54 (respectively, for κ-carrageenan, κ-carrageenan—pomegranate flesh and peel extracts)	-	Potential active intelligent food packaging material	[116]
κ-carrageenan—cassava starch	Improved thermal and mechanical properties High stiffness and water solubility Low swelling degree and water vapor permeability	TS (MPa): 19.23 ± 3.58, 25.88 ± 2.55 (respectively, for 100% κ-carrageenan, 75% κ-carrageenan—cassava starch) EB (%): 4.36 ± 0.90, 8.41 ± 1.71 (respectively, for 100% κ-carrageenan, 75% κ-carrageenan—cassava starch)	Inflection points in DTG curves for the κ-carrageenan (210 °C) and starch (334 °C) films	Potential active food packaging material	[13]
κ-carrageenan—cellulose nanocrystals	Improved mechanical, water, and UV barrier properties and thermal stability	TS (MPa): 38.33 ± 3.79, 52.73 ± 0.70 (respectively, for κ-carrageenan, κ-carrageenan—7% cellulose nanocrystals) EB (%): 21.50 ± 3.72, 25.83 ± 2.61 (respectively, for κ-carrageenan, κ-carrageenan—7% cellulose nanocrystals)	The first degradation, which occurred at 30–200 °C The second degradation at 230–400 °C	Potential active food packaging material	[117]
κ-carrageenan-gelatin—TiO_2_ NPs—anthocyanin	Improved mechanical properties and moisture resistance Changes in the freshness of the fish samples caused the films to change color	TS (MPa): 53.9 ± 0.6, 23.6 ± 2.2 (respectively for 3% κ-carrageenan-gelatin, 3% κ-carrageenan-gelatin—3% TiO_2_ NPs—anthocyanin) EB (%): 1.47 ± 0.05, 30.4 ± 0.2 (respectively, for 3% κ-carrageenan-gelatin, 3% κ-carrageenan-gelatin- 3% TiO2 NPs—anthocyanin)	The first degradation 170–200 °C Second degradation around 230–250 °C Third degradation around 460–480 °C	Potential smart and active packaging material	[118]
κ-carrageenan—honey bee pollen phenolic compounds	Increased physical properties and hydrophilicity Increased antioxidant and antiradical activity	TS (MPa): 24.60 ± 1.65, 35.97 ± 0.95 (respectively, for κ-carrageenan, κ-carrageenan—honey bee pollen phenolic compounds) EB (%): 69.91 ± 1.75, 78.64 ± 2.08 (respectively, for κ-carrageenan, κ-carrageenan—honey bee pollen phenolic compounds)	T_Onset_: 348 °C, 348 °C (respectively, for κ-carrageenan, κ-carrageenan—honey bee pollen phenolic compounds)	Potential edible films for beef	[119]
Cellulose					
Carboxymethyl cellulose—chitosan—ZnO NPs	Reduced water vapor permeability and increased antimicrobial activity	-	-	Potential active food packaging material for bread by reducing microbial growth	[18]
Carboxymethyl cellulose—guanidinylated chitosan enriched with TiO_2_ NPs	Improved thermal stability, mechanical, and UV barrier properties and antimicrobial activity	TS (MPa): 25.12 ± 1.43, 29.36 ± 1.88 (respectively, for carboxymethyl cellulose, carboxymethyl cellulose—guanidinylated chitosan enriched with 5% TiO_2_ NPs) EB (%): (respectively, for carboxymethyl cellulose, carboxymethyl cellulose—guanidinylated chitosan enriched with 5% TiO_2_ NPs)	The first mass loss around 100 °C The second mass loss of ca. occurred at 216–326 °C The third mass loss around 600 °C	Potential active food packaging material for excellent resistance to mass loss and spoilage of green bell pepper	[20]
Methylcellulose—jambolão (*Syzygium cumini*) skins extract	Improved mechanical and barrier performance Biodegradation of film in sea water in 2 days and soil in 15 days	TS (MPa): 16.10 ± 1.52, 21.4 ± 1.55 (respectively, for methylcellulose, methylcellulose film—50% jambolão extract) EB (%): 14.2 ± 2.0, 37.5 ± 2.0 (respectively, for methylcellulose, methylcellulose film—50% jambolão extract)	Tg (°C): 166.07, 135.97 (respectively, for methylcellulose, methyl-cellulose film—50% jambolão extract) Tm (°C): 174.37, 161.46 (respectively, for methylcelllose, methyl-cellulose film—50% jambolão extract)	Potential active intelligent food packaging for meat and aquatic products, where lipid oxidation occurs, and the pH modification is associated with food spoilage	[120]
Carboxymethyl cellulose (CMC)-starch	Improved mechanical properties, and water vapor barrier with the addition of CMC. Slight reduction in the thermal stability	TS (MPa): 50.2 ± 6.9, 32.6 ± 2.1 (respectively, for CMC, 80% CMC-20% starch) EB (%): 7.6 ± 2.2, 21.2 ± 4.3 (respectively, for CMC, 80% CMC-20% starch)	The first degradation at approximately 95 °C The second step of the thermal occurs between 145 °C and 160 °C. The third stage occurred in the range of 250–350 °C	Potential active food packaging material	[121]
Cellulose—ZnO NPs	Improved UV and oxygen barrier properties, thermal stability, and crystallinity Increased antimicrobial properties for *B. cereus*, *S. aureus*, *L. monocytogenes*, *E. coli*, *S. typhimurium*, and *V. parahaemolyticus*	TS (MPa): 141.70 ± 3.70, 126.61 ± 15.34 (respectively, for cellulose, cellulose—1% ZnO NPs) EB (%): 3.05 ± 0.34, 2.58 ± 0.73 (respectively, for cellulose, cellulose—1% ZnO NPs)	Minor weight loss of cellulose films at 50–55 °C Depending on the concentration of ZnONP, the thermal degradation was observed in the range of 270–330 °C	Potential antimicrobial food packaging material	[122]
Bacterial cellulose (BC)-carboxymethyl cellulose (CMC)-yeast	High water solubility antimicrobial activity against *E. coli*, *P. aeruginosa*, and *S. aureus* Enhanced shelf-life of orange and tomato coatings	TS (MPa): 17.02 ± 1.19, 2.23 ± 0.33 (respectively, for BC, BC-CNC-yeast) EB (%): 4.77 ± 0.56, 15.53 ± 0.84 (respectively, for BC, BC-CNC-yeast)	BC-CNC-yeast first degradation step at 90 °C BC first degradation step at 100 °C Second degradation step for BC-CNC-yeast started between 240 °C to 260 °C and continued until 330 °C BC cellulose skeleton degrades up to 300 °C	Potential edible food packaging materials	[123]
Agar					
Agar—melanin NPs	Improved UV-blocking, hydrophobicity, mechanical, water vapor barrier properties, and antioxidant activity	TS (MPa): 34.8 ± 0.7, 46.7 ± 1.7 (respectively, for agar, agar—0.5% melanin NPs) EB (%): 11.8 ± 0.7, 12.2 ± 0.9 (respectively, for agar, agar—0.5% melanin NPs)	Initial weight loss at 60–110 °C The next weight loss started at around 200 °C The maximum weight loss at 250 °C	Potential antioxidant active food packaging material	[21]
Agar—thermoplastic corn starch—glycerol	Improved barrier, tensile properties, and light transmittance Decreased water permeability and solubility	TS (MPa): 1.8 ± 0.2, 10.7 ± 2.1 (respectively, for thermoplastic corn starch, 60% agar—thermoplastic corn starch)	-	Potential active food packaging material	[22]
Agar—grey triggerfish skin gelatin—vine leaves ethanolic extract	Improved mechanical properties, thermal stability, and antioxidant activity	TS (MPa): 68.15 ± 1.20, 62.50 ± 1.10 (respectively, for gelatin-agar bilayer and gelatin-agar bilayer—5 mg/mL vine leaves) EB (%): 21.20 ± 1.91, 25.20 ± 1.10 (respectively, for gelatin-agar bilayer and gelatin-agar bilayer—5 mg/mL vine leaves)	Tg (°C): 65.15, 65.24 (respectively, for gelatin-agar bilayer and gelatin-agar bilayer—5 mg/mL vine leaves)	Potential active food packaging material	[23]
Agar—sodium alginate-SiO_2_ NPs	Improved mechanical properties, tensile strength, UV barrier properties, water resistance, and thermal stability Low minimum swelling degree and water solubility	TS (MPa): 45.18 ± 1.34, 74.68 ± 2.23 (respectively, for agar—sodium alginate, agar—sodium alginate-10 wt% SiO_2_ NPs) EB (%): 33.04 ± 0.40, 52.99 ± 1.65 (respectively, for agar—sodium alginate, agar—sodium alginate-10 wt% SiO_2_ NPs)	The first step of weight loss occurred at 50–150 °C The second stage of weight loss was 160–310 °C The third step, for temperature higher than 310 °C	Potential active food packaging material	[3]
Agar—maltodextrin bees wax	Improved tensile strength, Young’s modulus, contact angle, surface hydrophobicity, and mechanical properties Low water vapor permeability	TS (MPa): ~20, ~40 (respectively, for agar—maltodextrin bees wax, agar—maltodextrin bees wax-tween 80)	The first endothermic peak centered at 65 °C, the second melting peaks around 110 °C	Potential active food packaging material for higher water vapor resistance material	[124]
Agar—AgNPs	Antimicrobial activity against *L. monocytogenes* and *E. coli* Color and oxidative rancidity preservation of beef	-	-	Potential active food packaging material.	[125]
Agar—sugarcane Wax—butterfly pea flower extract	Visual color change in the presence of ammonia vapors and pH (2–12) Enhanced physical and mechanical properties	TS (MPa): 0.412 ± 0.016, 1.140 ± 0.172 (respectively, for agar—butterfly pea flower extract, agar—sugarcane wax—butterfly pea flower extract) EB (%): 69.000 ± 0.091, 46.000 ± 0.175 (respectively, for agar—butterfly pea flower ex-tract, agar—sugarcane wax—butterfly pea flower ex-tract)		Potential intelligent food packaging material for optical tracking of shrimp freshness	[126]
Pectin					
Pectin–polycaprolactone	Improved thermal stability, mechanical properties, barrier properties, and hydrophobic nature.	EB (%): ~1, ~20 (respectively, for pectin, pectin–polycaprolactone)	The first one, centered around 100 °C, is due to the loss of water; the second between 200 and 400 °C is attributed to the pyrolytic decomposition of macromolecular chains; and the third one is between 500 and 700 °C	Potential active food packaging material.	[24]
Pectin—copaiba oil nanoemulsions	Improved elongation at break, and antimicrobial activity against *S. aureus* and *E. coli*	TS (MPa): 41.8 ± 6.5, 12.4 ± 4.7 (respectively, for pectin, pectin—6% copaiba oil nanoemulsions) EB (%): 1.7 ± 0.1, 2.4 ± 0.5 (respectively, for pectin, pectin—6% copaiba oil nanoemulsions)	Tonset (°C): 215, 200 (respectively, for pectin, pectin—6% copaiba oil nanoemulsions)	Potential active food packaging material	[27]
Pectin—cocoa bean shell waste extract—ZnO-Zn-NPs	Improved thermal, oxidative stability, and oxygen barrier properties Decrease in oxygen transmission rate	-	Tmax (°C): 231 ± 1, 229 ± 1 (respectively, for pectin, pectin—5% cocoa bean shell waste extract—3% ZnO-Zn-NPs)	Potential active food packaging material	[25]
Pectin—pullulan	Improved water vapor barrier, UV barrier, mechanical properties, thermal stability, and surface hydrophobicity Protection against oxidation for food preservation	TS (MPa): 19.5 ± 2.8, 19.1 ± 2.6, 23.2 ± 2.4 (respectively, for pectin, pullulan, 30% pectin-70% pullulan) EB (%): 1.8 ± 0.3, 4.7 ± 0.3, 2.9 ± 0.9 (respectively, for pectin, pullulan, 30% pectin–70% pullulan)	The first step weight loss between 60 and 120 °C The second degradation step in the temperature range 150–240 °C The third step of degradation between 240 and 370 °C	Potential active food packaging material	[40]
Pectin-starch—TiO_2_ NPs	Improved mechanical, thermal stability and moisture, and UV barrier properties Decreased moisture content, solubility, and moisture uptake	TS (MPa): 22.34 ± 0.89, 26.16 ± 0.16 (respectively, for pectin-starch, pectin-starch—TiO_2_ NPs) EB (%): 12.96 ± 0.43, 8.12 ± 0.94 (respectively, for pectin-starch, pectin-starch—TiO_2_ NPs)	Tg (°C): 63.05 ± 1.2, 79.63 ± 0.42 (respectively, for pectin- starch, pectin-starch—TiO_2_ NPs) Tm (°C): 156.41 ± 0.30, 172.33 ± 0.65(respectively for pectin-starch, pectin-starch—TiO_2_ NPs)	Potential edible film	[127]
Pectin—kiwifruit (*Actinidia chinensis)* peel extract	Enhanced tensile strength and Young’s modulus Increased the shelf-life of chicken thigh by lower degree of lipid oxidation	TS (MPa): 42.30 ± 0.82, 21.65 ± 0.97 (respectively, for pectin, pectin—1.5% kiwifruit peel extract) EB (%): 10.77 ± 0.70, 20.32 ± 1.32 (respectively, for pectin, pectin-1.5% kiwifruit peel extract)		Potential active food packaging material	[128]
Pectin-agar—zinc sulfide NPs	Improved mechanical and UV barrier properties High antibacterial activity against *E. coli* and *L. monocytogenes*	TS (MPa): 50.3 ± 2.8, 47.4 ± 3.2 (respectively, for pectin-agar, pectin-agar—zinc sulfide NPs) EB (%): 4.7 ± 1.5, 9.9 ± 2.6 (respectively, for pec-tin-agar, pectin-agar—zinc sulfide NPs)	The first weight loss occurred at 50–110 °C with a maximum decomposition temperature of 55–60 °C The second weight loss was observed at 115–250 °C with a maximum decomposition temperature of ~220 °C The third weight loss appeared 250–340 °C with a maximum degradation around 300 °C	Potential active food packaging material	[129]
Alginate					
Sodium alginate—oregano essential oil	Antimicrobial activity against *L. monocytogenes*	-	-	Potential edible film for prolong the shelf-life of ham slices by reducing microbial growth	[32]
Alginate—sepiolite modified with myrtle berries extract	Improved elongation at break, tensile strength, water vapor, and UV barrier properties	TS (MPa): 38 ± 4, 87 ± 8 (respectively, for alginate, alginate—sepiolite modified with myrtle berries extract) EB (%): 3.8 ± 0.9, 5.6 ± 0.9 (respectively, for alginate, alginate—sepiolite modified with myrtle berries extract)	The first stage of weight loss 100 °C The second stage occurs in the temperature range of 110–160 °C The third stage occurs in the temperature range of 160–366 °C The fourth stage at temperatures above 366 °C	Potential active food packaging material	[28]
Sodium alginate-carboxymethyl cellulose—epigallocatechin gallate	Improved antimicrobial activity and lipid oxidation prevention	TS (MPa): 4.28 ± 0.69, 10.78 ± 2.15 (respectively, for sodium alginate, sodium alginate—carboxymethyl cellulose—1.6% epigallocatechin gallate) EB (%): 27.50 ± 2.08, 11.20 ± 1.57 (respectively, for sodium alginate, sodium alginate—carboxymethyl cellulose—1.6% epigallocatechin gallate)	-	Edible coatings for prolong the shelf-life of fresh pork by reducing lipid oxidation and microbial growth	[31]
Sodium alginate-pectin-citric acid—tartaric acid	Improved tensile strength, chemical resistivity, and thermal properties Nontoxic and biodegradable	TS (MPa): 18.38, 17.20 (respectively, for sodium alginate-citric acid, pectin—citric acid)	Tonset (°C): 99.8, 99.9	Potential edible packing film for food wrapping	[130]
Alginate—Zn-MgO NPs	Antibacterial activity against *L. monocytogenes* Moderate cytotoxicity of MgO NPs towards mammalian cells	-	-	Extend the shelf-life of Cold-Smoked Salmon by controlling *L. monocytogenes* growth Potential antimicrobial active food packaging material	[131]
Sodium alginate-cellulose nano whisker—copper oxide NPs	Antibacterial activity against *S. aureus*, Salmonella sp., *C albicans,* and *Trichodenna* spp. Increased antioxidant activity	-	-	Prevent microbial contamination in fresh cut pepper Potential active food packaging material	[132]
Alginate—aloe vera–garlic oil	Enhanced thermal and mechanical properties Increased UV barrier properties and antimicrobial properties Enhanced shelf-life of coated tomato	TS (MPa): 17 ± 0.71, 21.85 ± 1.22 (respectively, for alginate, alginate—2% aloe vera–5% garlic oil) EB (%): 10 ± 0.91, 41.55 ± 0.64 (respectively, for alginate, alginate—2% aloe vera–5% garlic oil)	The first stage of mass loss around 100 °C The second step of mass loss at 218 °C The 3rd stage of mass loss at 266 °C	Edible coating for tomato	[133]
Alginate—sulfur NPs	Enhance mechanical and water vapor barrier properties Antimicrobial activity against *L. monocytogenes*	TS (MPa): 58.5 ± 0.8, 63.8 ± 1.2 (respectively, for alginate, alginate—3% sulfur NPs) EB (%): 7.5 ± 0.1, 6.8 ± 0.9 (respectively, for alginate, alginate—3% sulfur NPs)	The initial weight loss of up to 100 °C The second step degradation occurred between 200 and 300 °C	Potential active food packaging material	[134]
Gums					
Gellan gum—xanthan gum-zinc oxide NPs	Improved tensile strength, thermal stability, and water and UV barrier properties Decreased contact angle and water vapor permeability	TS (MPa): 22.1 ± 0.9, 35.5 ± 1.2 (respectively, for gellan gum—xanthan gum, gellan gum—xanthan gum-5 wt% zinc oxide NPs) EB (%): 30.0 ± 1.5, 25.1 ± 1.1 (respectively, for gellan gum—xanthan gum, gellan gum—xanthan gum-5 wt% zinc oxide NPs)	Tg (°C): 69.9 ± 0.4, 74.8 ± 0.9 (respectively, for gellan gum—xanthan gum, gellan gum—xanthan gum-5 wt% zinc oxide NPs) Tm (°C): 217.0 ± 0.3, 219.3 ± 0.4 (respectively for gellan gum—xanthan gum, gellan gum—xanthan gum-5 wt% zinc oxide NPs)	Potential active food packaging material	[34]
Xanthan gum—PVA-red grape pomace	Improved mechanical strength, antioxidant and antimicrobial activity	-	-	Potential active food packaging material	[33]
Xanthan—curdlan	Improved tensile strength, water solubility, mechanical, and moisture barrier properties	TS (MPa): ~16, ~14, ~28 (respectively, for curdlan, xanthan, 50% xanthan-50% curdlan) EB (%): ~25, ~7, ~17 (respectively, for curdlan, xanthan, 50% xanthan-50% curdlan)	The first weight loss observed between 86.5 and 104.33 °C The maximum weight loss was observed between 294.3 and 319.04 °C	Potential active food packaging material	[135]
Gellan gum—purple sweet potato anthocyanins	Improved mechanical properties, water-resistant and antioxidant activity Reduced hydrophilicity, swelling properties, and water vapor transmission rates	TS (MPa): 1.2 ± 0.2, 8.9 ± 1.1 (respectively, for gellan gum, gellan gum—purple sweet potato anthocyanins) EB (%): 1.5 ± 0.9, 4.3 ± 1.2 (respectively, for gellan gum, gellan gum—purple sweet potato anthocyanins)	-	Potential intelligent food packaging material to detect the spoilage of protein-rich foods caused by bacteria growth	[136]
Gellan gum—agar-montmorillonite	Improved thermal stability, tensile strength, and rheological properties. Decreased water barrier properties and contact angle	TS (MPa): 29.9 ± 1.2, 44.0 ± 1.4 (respectively, for gellan gum— agar, gellan gum—agar-10% montmorillonite) EB (%): 29.5 ± 0.9, 19.9 ± 0.8 (respectively, for gellan gum-—agar, gellan gum—agar-10% montmorillonite)	Tg (°C): 70.2 ± 0.4, 77.1 ± 0.8 (respectively, for gellan gum—agar, gellan gum—agar-10% montmorillonite) Tm (°C): 198.4 ± 0.3, 214.2 ± 0.5 (respectively, for gellan gum—agar, gellan gum—agar-10% montmorillonite)	Potential active food packaging material	[87]
Tragacanth gum—PVA gallic acid	Improved tensile properties and water vapor transmission rate Enhanced hydrophobicity and thermal stability	TS (MPa): 15.3 ± 2.1, 45.7 ± 1.4 (respectively for PVA, tragacanth gum—PVA gallic acid) EB (%): 149.3 ± 16.2, 69.4 ± 25.1 (respectively, for PVA, tragacanth gum—PVA gallic acid)	Tg (°C): 43.3, 70.5 (respectively, for PVA, tragacanth gum—PVA gallic acid) Tm (°C): 192.7, 216.3 (respectively, for PVA, tragacanth gum—PVA gallic acid)	Potential active food packaging material	[137]
Lignin					
Lignin—gellan gum-hydroxyethyl cellulose	Improved thermal, mechanical, hydrophobic, and UV barrier properties and antioxidant activity Showed non-cytotoxic activities and antimicrobial activity	TS (MPa): 23.0 ± 1.1, 39.0 ± 0.8 (respectively, for gellan gum, lignin—gellan gum-hydroxyethyl cellulose) EB (%): 20.3 ± 0.4, 32.5 ± 0.4 (respectively, for gellan gum, lignin—gellan gum-hydroxyethyl cellulose)	Tg (°C): 149.2 ± 0.5, 156.9 ± 0.3 (respectively, for gellan gum, lignin—gellan gum-hydroxyethyl cellulose) Tm (°C): 205.6 ± 0.6, 216.0 ± 0.3 (respectively, for gellan gum, lignin—gellan gum-hydroxyethyl cellulose)	Potential active food packaging material	[93]
Alkali lignin-lignosulfonate—soy protein isolate	Improved mechanical, UV barrier, and thermal properties Decreased water vapor permeability	TS (MPa): 4.74 ± 0.34, 8.01 ± 0.89, 10.98 ± 1.02 (respectively, for soy protein, 10% lignosulfonate–soy protein, 10% alkali lignin—soy protein) EB (%): 126.33 ± 17.9, 79.95 ± 5.32, 7.45 ± 1.24 (respectively, for soy protein, 10% lignosulfonate—soy protein, 10% alkali lignin—soy protein)	The first weight loss 50–100 °C. The second weight loss occurred at around 300 °C	Potential active food packaging material	[36]
Lignin—nanocellulose	Enhanced oxygen permeability and UV barrier properties	-	-	Potential active food packaging material	[37]
Lignin—poly(lactic acid)	Enhanced mechanical and thermal properties Good antioxidant activity	TS (MPa): ~40, ~30 (respectively, for PLA, PLA—40% lignin) EB (%): ~15, ~2 (respectively, for PLA, PLA—40% lignin)	Tonset (°C): 323.6, 306.1 (respectively, for PLA, PLA—40% lignin) Tmax (°C): 330.2, 320.7 (respectively, for PLA, PLA—40% lignin)	Potential active food packaging material	[38]
Pullulan					
Pullulan-tempo cellulose nanofibrils—montmorillonite clay	Improved tensile strength, thermal stability, and water barrier properties and decreased moisture susceptibility	TS (MPa): ~35, ~5 (respectively, for pullulan, pullulan-tempo cellulose nanofibrils—5% montmorillonite clay)	Maximum decomposition temperature for pullulan and pullulan-tempo cellulose nanofibrils—montmorillonite clay film were around 98 °C and 308.27 °C	Potential active food packaging material	[39]
Pullulan—lysozyme nanofibers	Improved mechanical properties, thermal stability, and antioxidant activity Antibacterial activity against *S. aureus* and lysozyme-resistant bacteria	TS (MPa): 35.0 ± 4.4, 37.6 ± 2.2 (respectively, pullulan, pullulan—5% lysozyme nanofibers) EB (%): 6.63 ± 1.11, 1.84 ± 0.29 (respectively, pullulan, pullulan—5% lysozyme nanofibers)	Pullulan has a single weight loss step with initial and maximum decomposition temperatures of 250 and 300 °C Lysozyme nanofibers has a single-step degradation profile with maximum degradation temperature of 308 °C	Potential edible films for active packaging	[138]
Pullulan—egg white	Improved mechanical properties Film showed lower degradation speed	TS (MPa): 60.65, 329.48 (respectively, for pullulan, pullulan—egg white) EB (%): 1.43, 10.33 (respectively, for pullulan, pullulan—egg white)	Initial loss at 100 °C Final weight loss step at 270–450 °C	Potential edible films for active packaging	[139]
Pullulan-graphene—nanocellulose	Increased opacity, hydrophobicity, tensile strength, oxygen transmission rate, and water vapor transmission rate Antibacterial activity against *E. coli* and *S. aureus*	TS (MPa): ~7, ~20 (respectively, for pullulan—nanocellulose, pullulan-graphene—nanocellulose	-	Potential active food packaging material	[140]
Pullulan-curcumin—Ag NPs	Maintained the textural and physicochemical broiler meat for 14 days of storage attributes along with minimal oxidative rancidity	-	-	Potential active food packaging material	[141]
Pullulan-carboxylated cellulose nanocrystal-tea polyphenol	Enhanced water barrier properties, thermal stability, and tensile strength Improved UV barrier properties, antioxidant activity, and antimicrobial activity	TS (MPa): 25.28 ± 1.21, 34.49 ± 1.32 (respectively, for pullulan-carboxylated cellulose nanocrystal, pullulan-carboxylated cellulose nanocrystal-5% tea polyphenol) EB (%): 8.67 ± 0.54, 5.76 ± 0.25 (respectively, for pullulan-carboxylated cellulose nanocrystal, pullulan-carboxylated cellulose nanocrystal-5% tea polyphenol)	The first step of thermal degradation was 80–150 °C Maximum decomposition temperature at around 230–400 °C	Potential active food packaging material	[142]
Pullulan-chitin nanofbers-curcumin—anthocyanins	Antioxidant and antimicrobial activities Color change with pH	TS (MPa): 23.95 ± 5.57, 10.18 ± 4.37 (respectively, for pullulan, pullulan-chitin nanofibers-curcumin—anthocyanins) EB (%): 7.45 ± 2.66, 10.05 ± 6.83 (respectively, for pullulan, pullulan-chitin nanofibers-curcumin—anthocyanins)	Significant weight loss at temperatures between 250 and 400 °C	Potential active and intelligent food packaging material	[143]
Pullulan—propolis extract	Improved UV barrier and decreased transparency Enhanced antimicrobial activity mainly against yeast	TS (MPa): 24.62 ± 2.12, 14.42 ± 1.99 (respectively, for pullulan, pullulan—propolis extract) EB (%): 21.00 ± 0.92, 15.92 ± 1.51 (respectively, for pullulan, pullulan—propolis extract)	-	Potential active food packaging material	[144]
Curdlan					
Curdlan—PVA-thyme essential oil	Improved elongation at break, antioxidant activity, and antibacterial activity Decrease in water vapor permeability was lower	TS (MPa): ~9, ~12 (respectively, for curdlan, 4curdlan—1PVA-thyme essential oil) EB (%): ~90, ~180 (respectively, for curdlan, 4curdlan—1PVA-thyme essential oil)	The heat absorption peak of curdlan film is 309 °C When PVA is added, the heat absorption peak conversion temperature of the film is up to 342 °C	Increased shelf-life of chilled meat up to 10 days Potential active food packaging material.	[41]
Curdlan-Xanthan	High mechanical and moisture barrier properties was observed in the blend films with 5:5 and 4:6 ratios of xanthan and curdlan	TS (MPa): ~16, ~14, ~28 (respectively, for curdlan, xanthan, 50% xanthan-50% curdlan) EB (%): preparation of a novel curdlan/bacterial cellulose/cinnamon essential oil blending film for food packaging application 25, ~7, ~17 (respectively, for curdlan, xanthan, 50% xanthan-50% curdlan)	The first weight loss observed between 86.5 and 104.33 °C The maximum weight loss was observed between 294.3 and 319.04 °C	Potential active food packaging material	[135]
Curdlan-bacterial cellulose-cinnamon essential oil	Enhanced tensile strength, the crystallinity, and the thermal stability Reduced water vapor permeability, moisture content, and the lightness Good antibacterial activity and antioxidant capacity	TS (MPa): ~5, ~7 (respectively, for curdlan, curdlan-2% bacterial celllose-15% cinnamon essential oil) EB (%): ~70, ~80 (respectively, for curdlan, curdlan-2% bacterial celllose-15% cinnamon essential oil)	The first heat absorption peak of the films was observed around 40–110 °C Exothermic peak of curdlan films are around 270–300 °C Exothermic peaks of blending film were around 285 °C and 282 °C	Potential active food packaging material	[42]

The incorporation of NPs such as cellulose nanofibers, [105] cellulose nanocrystals [7], and ZnO [8] also improves the properties of starch. Tibolla et al. [105] developed a bio-nanocomposite film by using cellulose nanofibers isolated from the unripe banana peel by acid hydrolysis as reinforcement agents in a matrix of banana starch. The cellulose nanofibers incorporated into starch films showed high elongation at break (30.6%), good tensile strength (12.3 MPa), low moisture (13.66%), solubility in water (24.1%), and inferior UV/light transmission. Coelho et al. [7] formed a bio-nanocomposite film by embedding pomace pre-treated cellulose nanocrystals into a starch matrix by casting technique. The incorporation of cellulose nanocrystals reduced the water vapor permeability of the starch films from 7.5 ± 0.35 g × h·m·Pa^−1^ to 4.25 (1% cellulose nanocrystals) and 4.55 × 10^−7^ g × h·m·Pa^−1^ (1% cellulose nanocrystals). Films containing 5 to 15% cellulose nanocrystals were more opaque and degraded faster when exposed to light, while the mechanical, and barrier properties were unaffected.

The combination of cinnamon essential oil and TiO_2_ NPs was incorporated into a Sago starch matrix to form packaging for fresh pistachios [6]. The addition of essential oil into the starch matrix enhanced the permeability of starch films to oxygen and water vapor, while increasing the concentration of TiO_2_ NPs lowered the barrier properties. Further, the moisture content of starch films was reduced from 12.96% to 8.04%, and water solubility declined from 25% to 13.7%. Starch-based films have been developed to become smart packaging systems along with antioxidant activity and color changes at different pH as for the study by Ceballos et al. [145] on yerba mate extract. Extrusion and compression molding were used to make native or hydrolyzed starch and yerba mate extract films. The developed film was hydrophobic with an increased plasticizer effect and disintegrated after 10 weeks of soil burial.

In the recent study of Li et al. [82] lactic acid bacteria (probiotic) and sodium carboxymethyl cellulose were incorporated into starch to form an edible film. To boost the film’s probiotic activity, two lactic acid bacteria species (*Lactiplantibacillus plantarum* and *Pediococcus pentosaceus*) with high exopolysaccharide yield were used from a pickled water sample. The composite film’s antioxidant activity was greatly increased, with the highest activity of 48.1%. The water vapor and light transmission of the film were reduced, thus resulting in lipid oxidation deterioration and leading to increased shelf-life of banana.

#### 6.1.2. Chitosan

Chitosan is a nontoxic, biodegradable polycationic copolymer derived from chitin by deacetylation. It is insoluble in water and organic solvents but forms polycations in media with a pH less than 6.5. Chitosan is a highly researched biodegradable biopolymer due to its attractive properties, which include biocompatibility, film-forming ability, antioxidant activity, antimicrobial activity, mechanical properties, selective permeability to CO_2_ and O_2_, UV barrier properties, good optical properties, transparency, flexibility, and fat and oil resistance. A chitosan-based packaging system increases the emulsifying effect, the natural flavor, the texture setting, the deacidification, and the color stabilization of food products, thereby enhancing their quality, safety, and shelf-life. However, the limitation of pure chitosan in food packaging is found to be weakened water vapor barrier properties; thus, it is highly sensitive to moisture, and a film developed from chitosan is found to be brittle with low elasticity [4,10,96,109,110,146].

Chitosan has been blended with many biopolymers in studies to increase the relevant properties. The non-covalent bond formation between chitosan and alginate makes them one of the most compatible biopolymers for food packaging [147], while starch–chitosan is also considered a promising blend film [148]. Further, polymer blends with pectin make packaging material transparent with increased mechanical properties [149]. Some studies in recent years have demonstrated that the addition of NPs to the chitosan matrix increases the antimicrobial properties of the film while prolonging the shelf-life of the food product. A study by Kaewklin, Siripatrawan, and Suwanagul [111] shows that TiO_2_ NPs exhibited ethylene photodegradation while delaying the ripening process and enhancing the quality of the tomatoes. In this study, TiO_2_ NPs were incorporated into a chitosan matrix to form a packaging film. Paiva et al. [12] reinforced graphene oxide NPs into chitosan to form packaging bags by the solvent casting method. The film decreased water vapor permeability and increased tensile strength and Young’s modulus while prolonging the shelf-life of melon fruits.

To overcome the shortfalls of the pure chitosan natural plant extracts such as olive pomace [150], purple-fleshed sweet potato extract [151], apple peel [152], black soybean seed coat extract [153], and Chinese chive root extract [154] have been used, which exhibit good antioxidant and antimicrobial activity. Further, some of these plant extracts (soybean seed coat extract and purple-fleshed sweet potato extract) were studied for pH-sensing ability, thus forming a smart food packing material.

**Figure 4:**
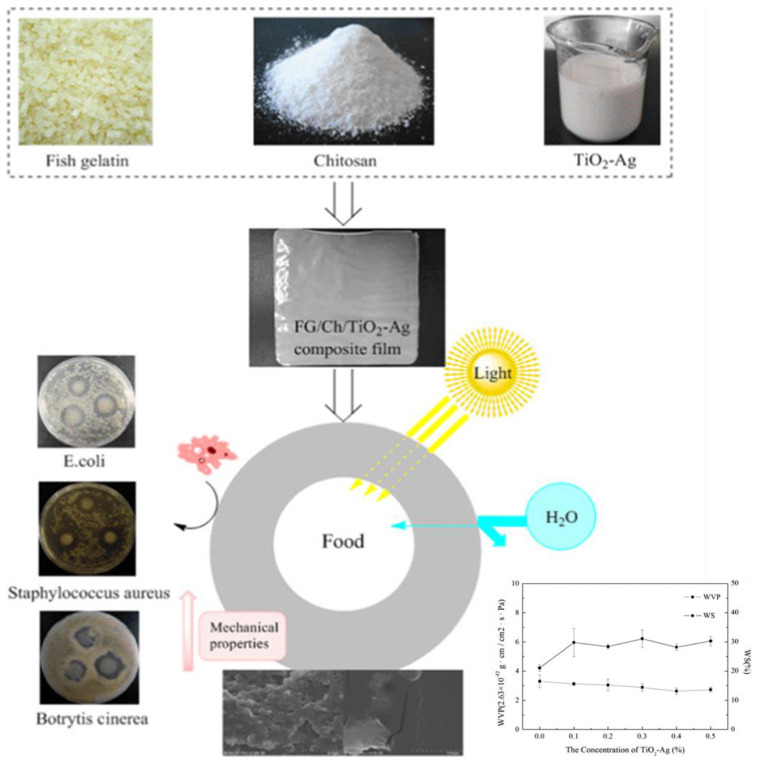
Development of chitosan–nano-silicon aerogel films incorporated with Okara powder by casting method. Reprinted/adapted with permission from Ref. [156]. 2020, Elsevier.

Glycerol/sorbitol is added to the chitosan matrix as plasticizers making the film less soluble in water, with increased optical property and less brittleness [10,155] Further, studies have been conducted using a combination of other biopolymers, NPs, and plant extracts together on the chitosan matrix. Jha [10] developed a packaging material with the combination of chitosan–starch–nanoclay and different ratios of grapefruit seed extract. The film containing 1.5% grapefruit seed extract showed increased mechanical (tensile strength of 19.6 MPa and 55.8% elongation at break), thermal, and water barrier properties. Further, this film showed a higher zone of inhibition against *A. niger* and high antifungal activity in stored bread at 25 °C for 20 days. Lin et al. [156] developed a functional food packaging material of chitosan–nano-silicon aerogel films incorporated with Okara powder by the casting method as shown in Figure 4. The produced films in general had increased flexibility, while the increased chitosan concentration of the film resulted in increased tensile strength. Further, the increased chitosan content led to a significant decrease in the water contact angle. The film also showed strong antibacterial activity against *E. coli* and *S. aureus*.

Further, a chitosan-based food packaging system with NPs was formed by Panariello, Coltelli, and Buchignani [157] with the incorporation of nanostructured chitin and cellulose. Here, chitosan and nanostructured chitin-based films were prepared in combination with cellulose by using the solution casting method.

#### 6.1.3. Carrageenan

Carrageenan is a linear, sulfated water-soluble polysaccharide that is extracted from red seaweeds belonging to the Rhodophyceae family [4,95]. It is widely used as a food additive. The commercial production of κ-carrageenan developed by CP Kelco is GENUGEL^®^, which is used as a thickening, stabilizing, gelling, and texturizing agent in food applications [13]. Carrageenan has a scope as a food packaging material due to its excellent film-forming ability, thermal stability, antibacterial properties, barrier properties, and biodegradability. However, it has limited application due to undesirable mechanical and water resistance properties [102]. The addition of other polysaccharides such as starch [14] significantly increases the physical, thermal, and mechanical properties of the film. Further to their studies, Sun et al. [158] designed an antioxidant and pH-responsive κ-Carrageenan-hydroxypropyl methylcellulose film with the incorporation of *Prunus maackii* juice. With the increasing *Prunus maackii* concentration, antioxidant activity reached 28.76%, and elongation at break was 48.64%. The lowest oxygen permeability was 1.63 cm^3^ mm m^−2^ day^−1^ atm^−1^, and the least water vapor permeability was 0.37 ± 0.01 g m^−1^ s^−1^ Pa^−1^ × 10^−12^. When the volatile base nitrogen content in the pork was 19.26 mg/100 g, the film turned from red to blue, indicating the monitoring of pork freshness.

Recently, further studies have been conducted using carrageenan biopolymer and nanofillers—carrageenan-CuSNPs [115], κ-carrageenan-*Zataria multiflora* extract—nanoclay [16], and κ-carrageenan-glycerol-cellulose nanocrystals [117]. Nanoclay, 1–3% *v*/*v* Zataria multiflora plant extract, and glycerol as a plasticizer were used to develop a biodegradable carrageenan nanocomposite film by using two different methods (adding glycerol before the formation of film-forming solution and after film-forming solution formation) [16]. The addition of glycerol to the carrageenan solution before the film formation solution increased tensile strength by 56% while lowering elongation at the break by 61%. All films were effective against *E. coli* and *P. aeruginosa*.

Additionally, Liu et al. [116] developed an active intelligent food packaging material using κ-carrageenan with the incorporation of pomegranate flesh or peel extracts. The incorporation of pomegranate flesh and or peel extracts enhanced the tensile strength from 24.73 MPa (pure κ-carrageenan film) to a maximum of 30.94 MPa and reduced water vapor permeability from 8.32 × 10^−11^ g m^−1^ s^−1^ Pa^−1^ (pure κ-carrageenan film) to a minimum of 3.47 × 10^−11^ g m^−1^ s^−1^ Pa^−1^. Furthermore, due to the abundance of anthocyanins, pomegranate-flesh-extract-containing films demonstrated pH-sensitive properties.

To develop a less expensive film, semi-refined carrageenan has been produced. However, they have poor water vapor permeability and relatively poor mechanical properties. Due to its inferior optical properties, it can be used for packaging applications such as food containers and cups. The mechanical and water barrier properties of semi-refined carrageenan film samples can be enhanced by photo-crosslinking with UV light to produce a low-cost food packaging material [17].

#### 6.1.4. Cellulose

Cellulose is the most abundant natural organic compound widely present in plants and bacteria. Cellulose is the agro-industrial waste that is mostly reused. The cellulose molecule (C_6_H_10_O_5_) n) has a linear ribbon-like conformation, and its compounds are bound together by the so-called β1-4, glycosidic bonds. It is widely used as raw materials for biodegradable films and edible films that are renewable, low cost, nontoxicity, biocompatible, biodegradable, odorless, tasteless, and chemically stable. Further, it has increased oxygen, hydrocarbon barrier properties, and water vapor permeability [91].

The most used cellulose derivatives in food packaging are methylcellulose (MC), hydroxypropyl methylcellulose (HPMC), hydroxypropyl cellulose (HPC), and carboxymethyl cellulose (CMC); however, due to their hydrophilic nature, they have poor water vapor barriers. In addition to the above demerits, cellulose has low mechanical strength and has opacity [17,30,121].

As per the study of Tavares et al. [121], they formed films through the solvent casting method by increasing the carboxymethyl cellulose concentration to form a neat starch film. There was an increase in the mechanical (elastic modulus was 14.5 times higher) and water vapor barrier properties of films by reducing the water vapor permeability by 56% (40% carboxymethyl cellulose incorporated film). However, there is a slight reduction in thermal stability from 294 °C for the pure starch film to 253 °C for the 40% carboxymethyl cellulose incorporated film [159]. Further, studies were carried out on carboxymethyl cellulose by [120] where glycerol, mucilage from *Dioscorea opposita*, and Ag NPs were incorporated into a carboxymethyl cellulose matrix by the casting method. With the decreasing concentrations of carboxymethyl cellulose in the film, tensile strength reduced from 18.30 MPa to 6.78 MPa, while the elongation at break increased from 38.52% to 62.33%. The water vapor permeability reduced from 25.06 g·mm/m^2^·d·kPa to 19.87 g·mm/m^2^·d·kPa. The film showed significant antibacterial activity against *S. aureus* and *E. coli*.

A pH-sensitive active film was produced from methylcellulose with the incorporation of anthocyanins present in jambolão (*Syzygium cumini*) skin by the casting technique. When compared to the pure methylcellulose film, the tensile strength improved from 16.10 MPa to 21.4 MPa (methylcellulose film + 50% jambolão extract), while elongation at break increased from 14.2% to 37.5% (methylcellulose film + 50% jambolão extract). As a result of the pH-sensitive structure of anthocyanins, color variations were observed in films when the pH was altered. Methylcellulose exhibited no radical scavenging activity, whereas increasing concentrations of jambolão jambolo extract increased radical scavenging activity. The films biodegraded in 2 days in sea water and 15 days in soil [120]. Additionally, studies have also been performed with the addition of a nanofiller to a cellulose matrix; examples are cellulose-lignin-cellulose nanocrystals [160], carboxymethyl cellulose-chitosan-ZnO NPs [18], and carboxymethyl cellulose-guanidinylated chitosan enriched with TiO_2_ NPs [20]. These packaging materials are considered potential active packaging materials with improved thermal stability, mechanical and UV barrier properties, and antimicrobial activity.

#### 6.1.5. Agar

Agar is a heterogeneous gelatinous polysaccharide extracted from marine red algae. The agar chain consists of D-galactopyranose and 3,6-anhydro-L-galactopyranose with alternating (1, 4) and (1, 3) linkages. Agar is insoluble in cold water and soluble in hot water, while it is stable in a different environment with low pH and high temperature [22,87]. It is used as a biodegradable film in food packaging because of its desirable film-forming ability, nontoxicity, stability in different environmental conditions, continuity, and transparency. However, its application in food packaging is limited due to poor water vapor barrier properties, mechanical properties, brittleness, thermal stability, and strong hydrophilic characteristics [4,21,23,87,92]. A considerable amount of research has been conducted on applications of agar in food packaging by the incorporation of reinforcement agents, for example, nanomaterials, other biopolymers, plasticizers, or antimicrobial agents.

The incorporation of other polymers such as maltodextrin–beeswax–liquid paraffin [124], maltodextrin–beeswax [161], starch [22], and gelatin [23] considerably increase the properties of agar. Fekete et al. [22] developed agar-based packaging films by casting or melt blending with a high glycerol concentration after agar was added to thermoplastic corn starch in a high concentration. The addition of agar to the starch matrix significantly increased the stiffness and strength of the film. Further, young’s modulus and tensile strength increased with the increasing concentrations of agar. Zhang et al. [161] developed an agar–maltodextrin–beeswax pseudo-bilayer edible film with different homogenizing speeds for mixing. The film was homogenized at a speed of 8000 rpm for 1 min and had the maximum tensile strength (20.57 MPa), young’s modulus (640.60 MPa), and contact angle (92.9°) and the minimum water vapor permeability (2.18 × 10^−12^ g m^−1^ s^−1^ Pa^−1^).

Melanin NPs [21], sodium alginate-nano-SiO_2_ [3], and montmorillonite-gellan gum [93] all improve the UV-blocking activity, hydrophobicity, mechanical properties, water vapor barrier, and thermal stability of agar, which makes them effective materials for food packing. Roy and Rhim [21] utilized melanin NPs isolated from sepia ink as the reinforcement agent for an agar-based packaging film. There was a decrease in the UV and visible light transmittance in the agar–melanin NPs film when compared to the pure agar film. The tensile strength (from 34.8 MPa to 39.8 MPa) and elongation at break (from 11.8% to 16.1%) of agar increased when 0.25% of melanin NPs was added. Lee et al. [87] combined another biopolymer gellan gum and montmorillonite nanoclay into an agar matrix to form a ternary nanocomposite film via the solution casting method. This film had improved thermal stability (119.4–174.7 °C) and tensile strength (29.9–44 MPa) upon the addition of montmorillonite. Furthermore, there was a decrease in the water barrier (1.9–1.7) and contact angle (56.8–49.4°) upon the incorporation of the montmorillonite nanoclay.

#### 6.1.6. Pectin

Pectin is a natural, renewable, and abundant polysaccharide in plant cell walls consisting of α (1–4) galacturonic acid monomers with different degrees of esterification [24]. It is an acid and water-soluble polymer that is used in industry as a stabilizing thickening, encapsulating, and gelling agent. Pectin’s beneficial characteristics, such as being biodegradable, renewable, cheap, gas permeable, and film-forming, make it a good material for edible films, biodegradable films, or gels used in food packing. However, pectin has certain characteristics that are not beneficial, such as negative mechanical properties, brittleness, low thermal stability, high solubility in water, and no antimicrobial properties [24,26,40,95,127].

Priyadarshi, Kim, and Rhim, [40] developed a hybrid biopolymer active food packaging material, pectin–pullulan, with different ratios of polymers through the solution casting method. The blend with the 50:50 ratio of pectin and pulullan exhibited the highest thermal stability, surface hydrophobicity, smallest water contact angle of 63.4°, and oil absorption value of 6.33 g m^−2^.

The thermal and oxidative stability and oxygen barrier properties of pectin have been improved by the addition of nanofillers into the matrix pectin–cocoa bean shell waste extract–ZnO-Zn-NPs [25]. Dash, Ali, and Das [127] also developed an edible film with lemon-waste pectin and sweet potato starch with TiO_2_ NPs by using the casting method. The film exhibited improved mechanical (tensile strength increased from 22.34 MPa to 26.16 MPa), moisture barrier, and UV barrier properties with the addition of TiO_2_ NPs. In recent years, studies have been performed on pectin modification such as pectin chemically modified with polycaprolactone, which reduces pectin’s hydrophilicity [24], pectin films activated by copaiba oil nanoemulsions that improve physicomechanical and antimicrobial properties [27], and thermoplastic pectin, which increases water resistance and mechanical properties [26].

#### 6.1.7. Alginate

Alginate is a natural polysaccharide extracted from brown algae, consisting of a (1–4) chain of a-L-guluronate and R-D-mannuronate. Alginate is regarded as a food safety additive by the FDA (US Food and Drug Administration) and EFSA (European Food Safety Authority). Commercially, alginate is used as a thickener, stabilizer, and gelling agent in foods such as deserts, sauces, and beverages [162]. Alginate can produce a strong insoluble polymer that has improved water barrier properties, mechanical properties, cohesiveness, stiffness, flavor maintenance, and slower fat oxidation. It has poor moisture barriers, and its hygroscopicity slows the dehydration of food [30,95]. Alginate is brittle, has poor water resistance, and is easily dissolved in water at room temperature [3]. Alginate packaging materials have been modified in recent years with the addition of other biopolymers such as sodium alginate–carboxymethyl cellulose–epigallocatechin gallate [31], and sodium alginate–pectin–citric acid–tartaric acid [130]; active agents such as sodium alginate–oregano essential oil [32] and alginate–sepiolite modified with myrtle berries extract [28]; and nanofillers such as alginate-Zn-MgO NPs [131] and sodium alginate-cellulose nano whisker-CuO NPs [132]. Singh et al. [130] developed a pectin (extracted from the waste pineapple shell)–sodium alginate (extracted from seaweed)-based film by crosslinking with citric acid and tartaric acid. The film was found to be a suitable edible packaging material through the studies of mice feed, plant growth substrate, and vermicomposting. Cheikh et al. [28] utilized the solution casting to make alginate nanocomposite films comprising sepiolite modified with polyphenol-rich myrtle berry extract. When compared to the control film, the hybrid films improved elongation at break (from 3.8% to 5.6%), tensile strength (from 38 MPa to 87 MPa), water vapor, and UV barrier properties. The films’ antioxidant activity was greatly improved and boosted as the myrtle berry extract content was increased.

#### 6.1.8. Gums

Gums are polysaccharides found in microbial production with a few different types. Arabic gums are found in the stems of various Acacia species, and it shows excellent film-forming ability, encapsulation properties, and unique emulsification. Xanthan gum is an exopolysaccharide synthesized by *Xanthomonas campestris*. Xanthan gum is used as a food stabilizer, thickener, and emulsifier. It forms a stable viscous solution in hot/cold water at different ranges of temperature and pH [95]. Gellan is a polysaccharide produced by *Sphingomonas elodea*. Gellan is used as a gelling agent, texturizer, and carrier for food additives in the food industry [95]. Gum biopolymers have a controlled viscosity, good biocompatibility, and low cytotoxicity. However, it has limited application in food packaging due to the high cost of production and low rheological, mechanical, and barrier properties. Recently, studies of xanthan gum biopolymers have been performed in developing xanthan gum–polyvinyl alcohol (PVA)-red grape pomace [33] and xanthan–curdlan [135] packaging materials, which were able to improve mechanical strength and antioxidant and antimicrobial activity. Lee et al. [87] developed ternary composite films from gellan gum–agar–montmorillonite via the solution casting method. The incorporated montmorillonite was able to improve thermal stability by 46.3%, tensile strength by 47.1%, and rheological properties. Further studies on the gellan gum intelligent food packaging material were performed by Wei et al. [136], which improved mechanical properties, water resistance, and antioxidant activity with the potential to detect the spoilage of protein-rich foods caused by bacteria growth. Finally, the studies of Rukmanikrishnan, Ismail, and Manoharan [34] were performed with the combination of two gum biopolymers and a nanofiller—gellan gum–xanthan gum–zinc oxide NPs—by using a solvent evaporation method. This combination improved tensile strength by 60.6%, thermal stability, and UV barrier properties. The water vapor permeability decreased by 39.7%, while moisture content values decreased by 38.0%.

#### 6.1.9. Lignin

Lignin is a complex phenolic compound that is abundantly found in the plant cell wall. It has good antioxidant properties and is a natural UV blocker. However, lignin has low mechanical and barrier properties. The combination with agar enhances the water vapor barrier and mechanical and thermal stability of the film while reducing the swelling ratio, transparency, and moisture content [4,160]. Limited studies have been performed on lignin biopolymers where lignin is combined with other biopolymers; lignin–gellan gum–hydroxyethyl cellulose [93] and alkali lignin–lignosulfonate–soy protein isolate [36] were tested to improve their UV barrier properties. Rukmanikrishnan, et al. [93] used the solvent casting process to make composite films using gellan gum, hydroxyethyl cellulose, and lignin (0, 1, 5, and 10 wt%). The addition of 10 wt% lignin increased the tensile strength of the film by 59.2%. This film showed 100% protection against UVB and 90% protection against UVA. The UV barrier properties of lignin were also observed in the studies of Zadeh, O’Keefe, and Kim [36] examining an alkali lignin–lignosulfonate–soy protein isolate film. Moreover, this film showed increased mechanical and thermal stability.

#### 6.1.10. Pullulan

Pullulan is a natural and biocompatible microbial polymer obtained from Aureobasidium pullulans. It has an alternation of α-(1,4) and α-(1,6) glycosidic bonds. Pullulan is currently used as a low-calorie component in food, coagulating agents, coating and wrapping material, and binders for fertilizers. It has many beneficial characteristics that can be used in food packaging such as being biodegradable, nontoxic, odorless, colorless, heat-sealable, water permeable, transparent, low in oxygen, oil permeable, and flexible. Further, it is also palatable and water-soluble, making it a suitable edible film material. However, it has high moisture sensitivity, affecting food packaging performance, in addition to low mechanical properties and brittleness [40,113].

The undesirable properties of pullulan were overcome in the study of Yeasmin et al. [39] by the addition of montmorillonite and tempo cellulose nanofibrils to a pullulan matrix. This film showed great optical transparency, moisture resistance, tensile strength (the highest 45.9 MPa was observed for 5 wt.% montmorillonite-containing films), and thermal properties. In addition to this study, Silva, Vilela, and Almeida [138] also developed a packaging material with pullulan–lysozyme nanofibers by a simple solvent casting technique from aqueous suspensions. This film showed improved mechanical properties (young’s modulus = 1.91–2.50 GPa), thermal stability, 77% DPPH scavenging activity, and antimicrobial properties. Studies on a potential edible film from pullulan—egg white were performed by Han, Liu, and Liu [139].

#### 6.1.11. Curdlan

Curdlan is nontoxic, biodegradable, colorless, odourless, has a high absorption/retention of moisture, can withstand extreme cold conditions, is insoluble in water, and is thermally stable. Nonetheless, it possesses weak mechanical properties. Studies of curdlan are extremely limited in food packaging applications, although it can be used as a suitable copolymer due to its characteristics. Zhang et al. [41] developed a packaging material based on curdlan-PVA-thyme essential oil. The curdlan: PVA film ratio of 4:1 had the highest tensile strength of 11.81 MPa and an elongation at break of 189.31%. The antioxidant properties of the film were improved by the addition of thyme essential oil, and the shelf-life of chilled meat was extended up to 10 days.

### 6.2. Protein-Based Biopolymers

**Figure 5:**
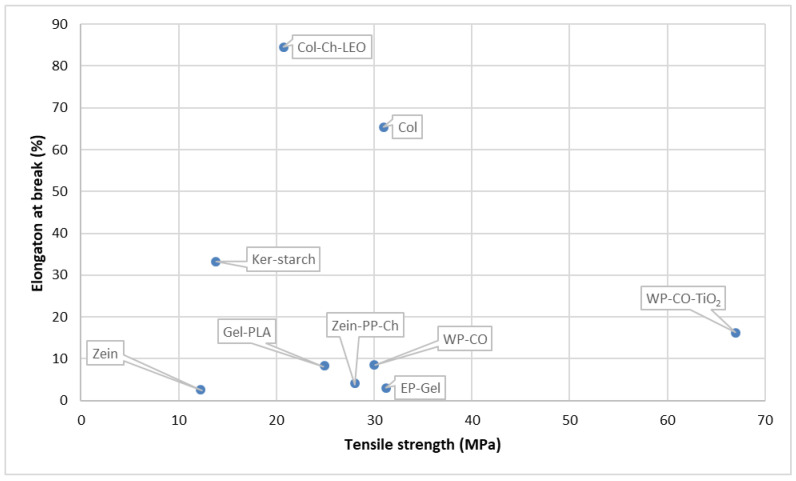
Elongation at break versus tensile strength for protein-based biopolymers. Abbreviation: Ker—Keratin, Col—collagen, LEO—lemon essential oil, Ch—chitosan, WP—whey protein, CO—corn oil, Gel—gelatin, PP—pomegranate peel extract, EP—Epigallocatechin gallate and laminated with PLA.

Protein biopolymers are made up of amino acid copolymers and can be divided into plant-origin proteins (e.g., gluten and soy) and animal-origin proteins (e.g., whey, collagen, and keratin). Protein biopolymers have many beneficial properties such as good mechanical properties, excellent gas barrier properties, good film-forming ability, nutritional value, and elasticity, thus making them suitable for food packaging applications. However, these proteins are hydrophilic, making them have poor water barrier properties. Protein-based biopolymers have potential applications in biomedicine and food packaging. Protein-based polymers including whey protein, gelatin, wheat gluten, corn, zein, and soy protein have been used to produce edible films in food packaging, improving their mechanical and barrier properties. Further, biopolymers such as keratin, casein, zein, gelatin, and soy protein play an important role in the preparation of various industrial products such as shopping bags, protection film, and sanitary products. The mechanical properties and other properties of protein biopolymers can be further enhanced by blending them with other biopolymers (protein/non-protein) or with other reinforcement agents as shown in Table 3 [55]. The mechanical properties of some of the studies depicted in Table 3 have been taken, and a scatter plot has been drawn for a better understanding of the properties, which can be seen in Figure 5. Protein biopolymers act as coating films in food packaging. WHEYLAYER and THERMOWHEY are European initiatives designed to develop coatings based on protein biopolymers. These initiatives developed oxygen barrier coatings for reusable multilayer packaging materials as an alternative to synthetic polymers [77].

#### 6.2.1. Whey Protein

Whey protein is a byproduct of the manufacturing of cheese. It is inexpensive, abundant, biodegradable, nutritious, film-forming, nutrient-rich, and has gas barrier properties. However, it has poor tensile strength and moisture resistance. Recent studies have been conducted in blended biopolymers such as whey protein–furcellaran–yerba mate–white tea extracts [52]. The incorporation of nanofiller such as whey protein–corn oil-TiO_2_NPs [50] and whey protein–chitosan nanofiber–nano-formulated cinnamon oil [163] has also been examined. Finally, the incorporation of active agents such as whey protein–nanoemulsions of orange peel (*Citrus sinensis*) essential oil has been studied [51].

**Figure 6:**
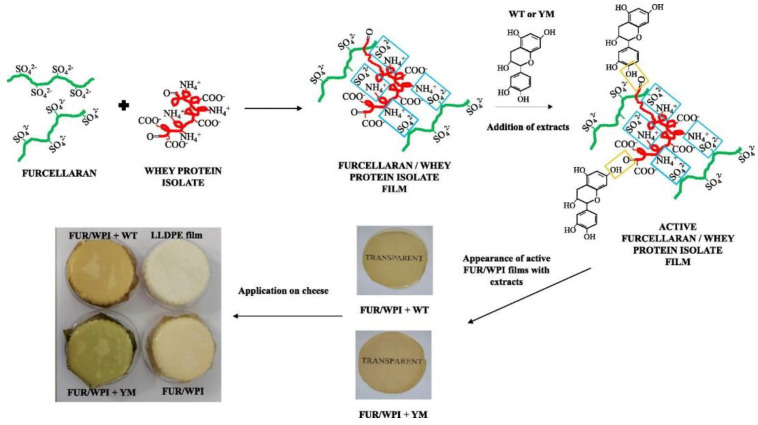
Development of whey protein–furcellaran-based edible film with the incorporation of yerba mate and white tea extracts [52]. Reprinted/adapted with permission from Ref. [52]. 2020, Elsevier.

Pluta-Kubica et al. [52] developed a whey protein films–furcellaran-based edible film with the incorporation of yerba mate and white tea extracts by using the casting method as shown in Figure 6. The permeability of water vapor, water content, solubility, modulus elasticity, and thermal stability of the film were all increased by yerba mate. During storage, the water content and activity of cheese packed in each type of biopolymer film reduced along with the coliform total bacterial count. Montes-de-Oca-Ávalos et al. [50] developed a TiO_2_NPs-incorporated corn oil–whey protein-based edible film with varying concentrations of whey proteins. The TiO_2_NPS-loaded bio-nanocomposite film had the highest elastic modulus (19.2 MPa), Young’s modulus (19.4 MPa), and elongation at break (119%).

#### 6.2.2. Gelatin

Gelatin is a renewable, sustainable protein source that is mostly found in the skin and bones of an animal. Collagen, in its natural form, has little use for application. Therefore, one chooses to extract the gelatin present in its composition for use. To obtain the gelatin, it is necessary for the collagen to undergo a hydrolysis process (acidic, alkaline, or enzymatic), associated with high temperatures to break the covalent bonds, releasing the gelatin molecules through denaturation of the helix triple. After cooling the solution, the chains absorb the water, forming gelatin. The two types of collagen (A and B) can be obtained from partial hydrolysis or thermal degradation of collagen. It is currently used in the food, pharmaceutical, and photographic industries due to its nontoxicity, renewability, biodegradability, and excellent film-forming ability. Furthermore, gelatin is biocompatible, adhesive, abundantly available, flexible, and transparent. It also is cheaper to manufacture while it has excellent water, aroma, and oxygen barrier properties. However, it is not suitable as a food packaging material alone due to its poor mechanical properties and processability [4,23,43,44]. Recent research has examined the blending of different biopolymers, such as Gelatin–PLA [43], Gelatin–chitosan-3-phenylacetic acid [164], and Gelatin–agar bilayer vine leaves [23]. The studies of Nilsuwan, Guerrero, and Caba [43] focused on developing a bilayer fish gelatin film incorporated with epigallocatechin gallate and laminated with PLA by thermo-compression molding. These films had high lipid oxidation retardation ability and thus can be used for the packaging of high-lipid-content foods. Ge et al. [165] also developed a green nanocomposite film with pH sensitivity and antioxidant activity using gelatin–chitin nanocrystals–anthocyanins that can be used for the freshness monitoring of high-protein foods. In addition, the bio-nanocomposite Gelatin–grapefruit seed-TiO_2_ NPs was developed by Riahi et al. [44] via the solution casting method. The film had improved mechanical properties, water contact angle, and antimicrobial and antioxidant activity, while it prevented UV light transmission completely.

#### 6.2.3. Soy Proteins

Soy proteins are abundant, renewable, and highly biodegradable proteins. Soy proteins consist of a high amount of polar amino acids such as cystine, arginine, lysine, and histidine. Thus, it has improved the mechanical strength, oxygen and lipid barrier, high water vapor permeability, and thermal properties in addition to flexibility, low cost, sustainability, biocompatibility, film-forming capacity, smoothness, and transparency. However, soy proteins have low water resistance, low thermoplasticity, brittleness, low mechanical properties, low film gloss, and low tensile strength. Soy proteins are available in three types—soy flour, soy protein concentrate, and soy protein isolate. Soya protein isolates are easily biodegradable but have poor plasticity, brittleness, and water vapor permeability [46,95].

#### 6.2.4. Zein

Zein is the main protein of corn endosperm and the chief byproduct of the bioethanol industry. It is a polyamine that has a high content of hydrophobic amino acids. Zein is soluble in ethanol and insoluble in water. Zein has great qualities for film-forming in the food packaging industry, such as hydrophobicity, antimicrobial potential, antioxidant activity, adhesive film-forming ability, and extreme resistance to moisture and oxygen. Further, Zein is also considered a safe material for the food system by the Food and Drug Administration (FDA). However, it breaks easily and has poor processability, mechanical properties, elongation at break, and thermal properties. Thus, it is unable to be used as a packaging material in its pure form. Plasticizers such as linoleic acid, palmitic acid, oleic acid, poly (ethylene glycol) (PEG), poly (propylene glycol) (PPG), poly (tetramethylene glycol) (PTMG), and glycerol has been added to zein films to increase brittleness, elasticity, and flexibility [46,95]. Recently, the properties of zein proteins have been enhanced by combining polymer blends and nanoparticles; studies have shown that the following combinations improve the properties: Zein–potato starch–chitosan NPs incorporated with curcumin [166], zein–chitosan–cinnamodendron dinisii Schwanke essential oil [55], zein–sodium alginate-TiO_2_ NPs-betanin [56], zein–pomegranate peel extract–chitosan NPs [167], and zein-TiO_2_ nanofibers [54]. Xin et al. [166] developed a zein–starch-based film with the incorporation of curcumin-loaded chitosan NPs by using the solution casting method. The highest tensile strength (13.1 MPa) and elongation at break (50.3%) were observed in the films with the lowest zein concentration (30%). These films were able to prolong the Schizothorax prenati fillets’ shelf-life up to 15 days. On the other hand, Amjadi, Almasi, and Ghorbani [56] used electrospinning technology to create novel nanofibers based on zein–sodium alginate integrated with TiO_2_ NPs and betanin for food packaging. The film showed acceptable mechanical properties, high surface hydrophobicity, and a DPPH scavenging activity of 64.42%. It also showed antimicrobial activity against *S. aureus* and *E. coli*.

#### 6.2.5. Keratin

Keratin is a natural protein found in bird feathers, wool, or other natural resources. Keratin is a biodegradable, biocompatible, and hydrophobic compound with greater absorption properties. However, keratin has very poor mechanical properties, making it not a suitable packaging material to be used in its pure form [168]. A limited number of studies have been performed on keratin-based food packaging materials. In recent years, Ramirez et al. [58] developed a keratin–citric acid-based food packaging material by using the casting method. This film showed an improved biocidal effect and a 600% elongation at the break. It can prolong the shelf-life of carrots when compared to commercial film. Further, Ramakrishnan et al. [59] also designed a keratin–glycerol-based biodegradable packaging material from keratin extracted from chicken feathers. The best mechanical and thermal properties are found in keratin films with 2% glycerol. Biodegradability tests have shown that all produced bioplastics films are biodegradable.

#### 6.2.6. Collagen

Collagen is a naturally abundant, biocompatible, and biodegradable protein found in animals. It is industrially produced from the skin and bones of swine, cattle, and fish skin. Collagen has a great film-forming ability, antioxidant properties, moisture and oxygen barrier, and structural integrity. However, its high water vapor transmission rates and poor mechanical properties give collagen limited applications in the food packaging industry. Thus, very limited studies have been performed on collagen food packaging [169]. Jiang et al. [60] fabricated a food packaging material with a collagen matrix by using the solvent casting method, where the chitosan–lemon essential oil NPs were incorporated. The film had improved tensile strength, elongation at break from 65.41% to 104.34% (30% chitosan–lemon essential oil NPs), and reduced oxygen permeability from 0.57 cm^3^ mm m^−2^ d^−1^ kPa^−1^ to 0.39 cm^3^ mm m^−2^ d^−1^ kPa^−1^ (30% chitosan–lemon essential oil NPs). With regard to the shelf-life study, when the pork was stored at 4 °C for 21 days, films significantly prevented lipid oxidation, reduced microbial multiplication, and prolonged the deterioration of pork.

### 6.3. Aliphatic Polyesters

**Figure 7:**
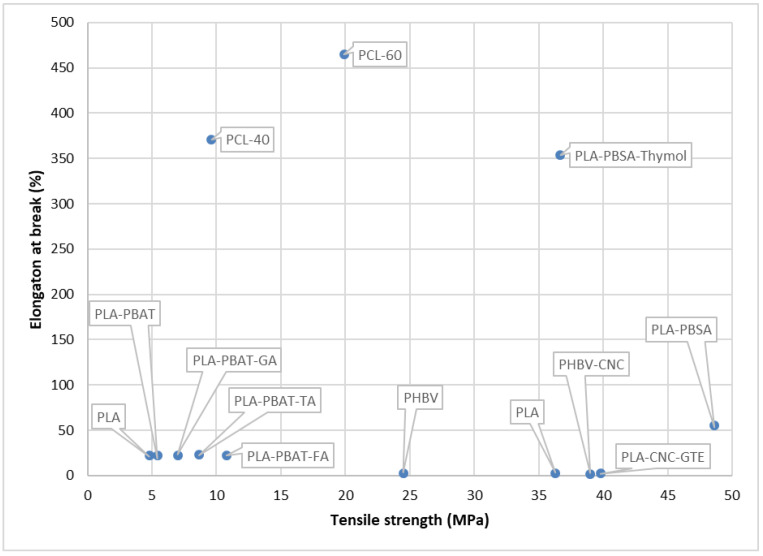
Elongation at break versus tensile strength for aliphatic polyester biopolymers. Abbreviation: CNC—cellulose nanocrystals, GTE—green tea extract, TA—tannic acid, GA—gallic acid, FA—ferulic acid.

Aliphatic polyesters are biopolymers composed of repeating structures that, upon degradation, produce metabolites such as poly(beta-hydroxy alkanoate)s and poly(alpha-hydroxy alkanoate)s. They are easily biodegradable because of the presence of ester bonds in the soft chains making them sensitive to hydrolysis. Aliphatic polyesters have been used as commercial products as an alternative to synthetic properties due to their similar properties; however, they lack mechanical and thermal properties [92]. Some of the aliphatic polyesters that are used in food packaging are PLA, PBAT, polyhydroxyalkanoate (PHA), polybutylene succinate (PBS), polyhydroxybutyrate (PHB), and polycaprolactone (PCL), and their properties are shown in Table 4. The mechanical properties of some of the studies depicted in Table 4 have been taken, and a scatter plot has been drawn for a better understanding of the properties, which can be seen in Figure 7.

Aliphatic polyesters amount for most of the bioplastics, which amount to 2.11 million tonnes of global production in 2020, out of which the highest market segment is the packaging market, which accounts for 47% (0.99 million tonnes). As for the European Bioplastic [78] PBAT, PLA, PBS, and PHA are currently in use in the rigid and flexible packaging marketing sector.

#### 6.3.1. Poly Lactic Acid (PLA)

Poly lactic acid (PLA) has become one of the most significant commercial polymers that are biodegradable and bio-based thermoplastics. PLA is made up of alpha-hydroxy acids, which include polyglycolic acid or polymandelic. PLA is derived by depolymerization of the lactic acid monomer obtained from sugar cane, corn starch, or tapioca. Companies such as Ingeo (Nature Works, Plymouth, MN, USA), PURAC (PURAC Co., Rayong, Thailand), BIOFRONT (Teijin, Tokyo, Japan), HiSun (Revoda, Stoney Creek, ON, Canada), and Pyramid (Tate and Lyle, Pinckneyville, Denmark) manufacture biodegradable PLA films. These produced biopolymers are used in a wide variety of industrial applications such as disposable household items (drinking cups, cutlery, trays, food plates, and food containers), food packaging, waste bags, shopping bags, agriculture (soil retention sheeting and agriculture films), drug delivery systems, biomedical devices disposable garments, feminine hygiene products, and diapers [77]. PLA is used in commercial food packaging for manufacturing caps (PLA blends), coffee capsules/pouches (PLA/PHB), shopping/waste bags (Blends of PLA/PHA/PBAT), clear films for fruits and vegetables (PLA/Blends of PLA/Bio-PET), and teabags (PLA blends) [170].

**Table 3:** Applications of protein biopolymers in food packaging.

Packaging Material	Characteristics of Food Packaging System	Mechanical Properties	Thermal Properties	Application	Reference
Gelatin					
Gelatin-grapefruit seed (GSE)—TiO_2_ NPs	Improved mechanical properties, water contact angle, and antioxidant activity Decreased water vapor permeability and UV light transmittance. Antibacterial activity against *E. coli* and *L. monocytogenes*	TS (MPa): 60.6 ± 1.1, 57.9 ± 1.3, 63.4 ± 1.5, 61.5 ± 1.7, 58.3 ± 1.9, 55.2 ± 1.6 Gel, (respectively for Gel/GSE, Gel/GSE/0.5%TiO_2_, Gel/GSE/1%TiO_2_, Gel/GSE/2%TiO_2_, Gel/GSE/5%TiO_2_) EB (%)10.6 ± 0.8, 12.7 ± 1.2, 9.6 ± 1.7, 10.4 ± 2.0, 12.5 ± 1.1, 13.3 ± 1.9 (respectively, for Gel/GSE, Gel/GSE/0.5%TiO2, Gel/GSE/1%TiO2, Gel/GSE/2%TiO2, Gel/GSE/5%TiO2)	Initial weight loss at 80–120 °C, subsequent degradation varied between 200 and 300 °C, and third step of weight loss around 320 °C	Potential active food packaging material	[44]
Gelatin—PLA	Improved mechanical and UV–visible light barrier properties Low water vapor and oxygen permeability	TS (MPa): 24.90 ± 5.59, 31.21 ± 2.88 (respectively, for Gelatin-PLA and Epigallocatechin gallate, laminated with PLA and emulsified with gelatin) EB (%): 8.27 ± 3.26, 11.83 ± 3.05 (respectively, for Gelatin-PLA and Epigallo-catechin gallate, laminated with PLA and emulsified with gelatin)	-	Control lipid oxidation and increased shelf-life of fried salmon skins up to 30 days. Suitable active packaging material for high-lipid-content foods.	[43]
Gelatin-chitosan-3-phenylacetic acid	Improved thermal stability, water stability, and water vapor permeability Antimicrobial activity against *S. enterica* and *S. aureus*		The first weight loss occurred around 75–150 °C A major loss occurred at 200–300 °C	Potential active food packaging material	[164]
Gelatin-agar bilayer—vine leaves	Improved mechanical properties and antioxidant and amicrobial activity	TS (MPa): 68.15 ± 1.20, 62.50 ± 1.10 (respectively, for Gelatin-agar bilayer and Gelatin-agar bilayer—5 mg/mL vine leaves) EB (%): 21.20 ± 1.91, 25.20 ± 1.10 (respectively, for Gelatin-agar bilayer and Gelatin-agar bilayer—5 mg/mL vine leaves)	Tg (°C): 65.15, 65.24 (respectively, for Gelatin-agar bilayer and Gelatin-agar bilayer—5 mg/mL vine leaves)	Potential active food packaging material	[23]
Gelatin-oxidized chitin nanocrystals (Ch)—black rice bran anthocyanins (BACNs)	Improved UV–vis light barrier and antioxidant activity	TS: 9.44 ± 0.29, 2.53 ± 0.12 (respectively, for BACNs-Ch0 and BACNs-Ch100) EB (%): 115.33 ± 3.06, 141.67 ± 3.06 (respectively, for BACNs-Ch0 and BACNs-Ch100)	-	Monitor the freshness of shrimp and hairtail by visible color changes. Potential intelligent packaging material for freshness monitoring of high protein foods	[165]
Gelatin-carrageenan—carbon dots	Enhanced mechanical properties High antioxidant activity	TS (MPa): 52.8 ± 6.3, 81.2 ± 5.3 (respectively, for gelatin-carrageenan and gelatin-carrageenan-10% carbon dots) EB (%): 3.9 ± 1.1, 6.4 ± 0.6 (respectively, for gelatin-carrageenan and gelatin-carrageenan—10% carbon dots)	The first weight loss at 55−110 °C The second thermal degradation from 125 °C to 280 °C The third thermal degradation from 285 °C to 350 °C	Potential active food packaging material	[171]
Gelatin-carrageenan-shikonin—propolis	Excellent pH (2–12) responsive color-changing properties Enhanced UV barrier properties Monitored the freshness of packaged milk	TS(MPa): 43.9 ± 2.3, 41.7 ± 3.0 (respectively, for Gelatin-Carrageenan and Gelatin-carrageenan-shikonin—propolis) EB (%): 3.2 ± 0.2, 3.6 ± 0.1 (respectively, for Gelatin-Carrageenan and Gelatin-carrageenan-shikonin—proplis)	Three step degradation between 290 and 350 °C	Potential intelligent food packaging material	[172]
Gelatin—tea polyphenol/ε-poly (L-lysine)	High hydrophobicity and UV barrier properties Excellent antibacterial activity and antioxidant activity	-	-	Potential active food packaging material	[173]
Keratins					
Keratin—citric acid	Improved biocidal effect, elongation value, and transparency	TS(MPa): 1.49 ± 0.80 EB (%):138 ± 21	First stage of weight loss 60 °C for pure keratin and 80 °C for keratin—citric acid The second stage at 224 °C for pure keratin and 195 °C for keratin—citric acid	Increased shelf-life of carrot. Active packaging material suitable for food preservation	[58]
Keratin—glycerol	Improved mechanical and thermal properties. Fully biodegradable according to biodegradability test	TS(MPa): 9.59, 0.0409 (respectively, for 15 wt% glycerol-sugar palm starch film, 10 wt% keratin bioplastic film)	-	Potential active food packaging material	[168]
Feather keratin—dialdehyde carboxymethyl cellulose	Increased UV barrier properties and transmittance Reduced moisture sensitivity	TS(MPa): 17.6 ± 3.0, 30.8 ± 4.6 (respectively, for keratin, keratin—dialdehyde carboxymethyl cellulose) EB (%): 4.0 ± 0.9, 0.7 ± 0.4 (respectively, for keratin, keratin—dialdehyde carboxymethyl cellulose)	-	Potential edible food packaging material	[59]
Keratin—starch	Improved mechanical properties decayed over 20% of the original mass after 12 days of soil burial	TS(MPa): 8.3 ± 0.2, 13.8 ± 0.2 (respectively, for starch-keratin 20:0, starch-keratin 20:5) EB (%): 19.7 ± 0.1, 33.3 ± 0 (respectively, for starch-keratin 20:0, starch-keratin 20:5)	-	Potential active food packaging material	[174]
Keratin–gelatin–glycerin–curcumin	Enhanced mechanical properties Antibacterial activity against *S. aureus* and *E. coli*	TS(MPa): 12.45, 13.73 (respectively, for 7% keratin–10% gelatin–1% curcumin, 7% keratin–10% gelatin–2% glycerin–1% curcumin)	The initial degradation weight loss occurs between 25 °C and 130 °C The second degradation step is observed in the temperature range of 130–400 °C The third step of degradation occurs between 400 and 800 °C	Potential active food packaging material	[175]
Whey proteins					
Whey protein–furcellaran–yerba mate–white tea extracts	Improved water vapor permeability, water content, solubility, tensile strength, mechanical properties, and thermal stability	TS(MPa): 1.36 ± 0.32, 1.31 ± 0.20 (respectively, for whey protein–furcellaran, whey protein-–furcellaran–yerba mate–white tea extracts) EB (%): 25.99 ± 3.32, 25.13 ± 2.79 (respectively, for whey protein–furcellaran, whey protein–furcellaran–yerba mate–white tea extracts)	Peak temperature (Tm) (°C) (1st transition endothermic): 218.2 ± 1.1, 219.4 ± 2.3 (respectively, for whey protein–furcellaran, whey protein–furcellaran–yerba mate–white tea extracts)	Potential edible film for cheese packaging with decreased microbial growth and water content	[52]
Whey protein-corn oil—TiO_2_ NPs	Improved of mechanical and tensile properties	TS(MPa): 8.62 ± 0.59, 16.24 ± 0.29 (respectively, for 2.5% whey protein–corn oil–0% TiO_2_ NPs, 2.5% whey protein–corn oil–0.5% TiO_2_ NPs) EB (%): 30 ± 8, 67 ± 7 (respectively, for 2.5% whey protein–corn oil–0% TiO_2_ NPs, 2.5% whey protein–corn oil–0.5% TiO_2_ NPs)	The first stage of weight loss 50 to 110 °C The second stage of weight loss 120–220 °C The third stage of weight loss 250–340 °C Finally stage of weight loss 350 to 500 °C	Potential active food packaging material for cheese packaging	[50]
Whey protein–chitosan nanofiber–nano-formulated cinnamon oil	Improved UV barrier properties and antibacterial activity Decrease in water solubility and the water vapor permeability Slight reduction in tensile strength	TS(MPa): 4.09 ± 0.38, 3.41 ± 0.47 (respectively, for whey protein, whey protein–chitosan nanofiber–nano-formulated cinnamon oil) EB (%): 77.21 ± 0.49, 35.57 ± 5.85 (respectively, for whey protein, whey protein–chitosan nanofiber–nano-formulated cinnamon oil)		Potential active food packaging material	[163]
Whey protein isolate-coated multilayer film	Improved oxygen and water vapor permeability	TS(MPa): 45.80 ± 1.53, 33.57 ± 0.93 (respectively, for polyethylene terephthalate–whey protein isolate, low-density polyethylene–linear low-density polyethylene, polyethylene terephthalate–whey protein isolate, low-density polyethylene–linear low-density polyethylene–aluminum oxide) EB (%): 84.10 ± 14.67, 60.56 ± 4.94 (respectively, for polyethylene terephthalate–whey protein isolate, low-density polyethylene–linear low-density polyethylene, polyethylene terephthalate–whey protein isolate, low-density polyethylene–linear low-density polyethylene–aluminum oxide)		Preservation of physicochemical and sensory properties of frozen marinated meatloaf up to 6 months Potential frozen food packaging material	[53]
Whey protein—nanoemulsions of orange peel (*Citrus sinensis*) essential oil	Improved water barrier properties and antioxidant and antimicrobial activities	TS(MPa): 2.64 ± 0.62, 1.76 ± 0.44 (respectively, for whey protein, whey protein—5% of nanoemulsions of *Citrus sinensis*) EB (%): 11.40 ± 1.68, 18.65 ± 1.78 (respectively, for whey protein, whey protein—5% of nanoemulsions of *Citrus sinensis*)	-	Suitable active food packaging material for the preservation of food against oxidation and microbial spoilage	[51]
Whey protein isolate–polyvinyl alcohol–nano-silica	Improved water barrier properties and tensile strength	TS(MPa): 7.13, 10.2 (respectively, for whey protein isolate–polyvinyl alcohol, whey protein isolate–polyvinyl alcohol–4% nano silica)	Tg (°C): 19, 26 (respectively, for whey protein isolate–polyvinyl alcohol, whey protein isolate–polyvinyl alcohol–4% nano silica)	Potential active food packaging material	[176]
Zein					
Zein–potato starch–chitosan NPs incorporated with curcumin	Improved mechanical and barrier properties. High oxidation resistance and relative release efficiency	TS(MPa): 7.9 ± 0.8, 13.1 ± 2.3 (respectively, for zein–potato starch, zein–potato starch–chitosan NPs incorporated with curcumin) EB (%): 19.1 ± 1.6, 50.3 ± 4.1 (respectively, for zein–potato starch, zein–potato starch–chitosan NPs incorporated with curcumin)	-	Delayed physicochemical changes in *Schizothorax prenati* fillets and prolonged shelf-life up to 15 days. Potential bioactive packaging material for *Schizothorax prenati* fillets	[166]
Zein–chitosan–cinnamodendron dinisii schwanke essential oil	Improved antioxidant activity and antimicrobial activity	-	The endothermic peaks at −7.8 °C and −6.2 °C (respectively, for zein, zein–chitosan–*Cinnamodendron dinisii* schwanke essential oil)	Stabilizing deterioration reactions and preserving the color of ground beef	[55]
Zein-sodium alginate-TiO_2_ NPs-betanin	Improved mechanical properties, high surface hydrophobicity, antioxidant and antibacterial activity No in-vitro cell cytotoxicity	TS(MPa): 2.01 ± 0.26, 12.62 ± 1.24 (respectively, for zein–sodium alginate, zein–sodium alginate-TiO_2_ NPs–betanin) EB (%): 10.74 ± 2.11, 40.49 ± 3.72 (respectively, for zein–sodium alginate, zein–sodium alginate-TiO_2_ NPs–betanin)	-	Potential active food packaging material	[56]
Zein–pomegranate peel extract–chitosan NPs	Improved thermal stability and antimicrobial activity against *L. monocytogenes*	TS(MPa): 12.22 ± 1.2, 28 ± 1.06 (respectively, for zein, zein–pomegranate peel extract–chitosan NPs) EB (%): 2.6 ± 0.22, 4.1 ± 0.21 (respectively, for zein, zein–pomegranate peel extract–chitosan NPs)	The initial stage happened between 100 and 150 °C. The second stage of weight loss occurred at 200–250 °C	Restricted microbial growth in pork sample. Potential antimicrobial active food packaging material	[167]
Zein-TiO_2_ nanofibers	Improved thermal properties and ethylene absorption capability No significant differences in water contact angles	-	Zein nanofibers (0% TiO_2_) presented a one-step weight loss which peaked at approximately 240–390 °C	Potential active food packaging material	[54]
Zein–catechin-loaded β-cyclodextrin metal	Decreased water vapor permeability and swelling degree Increased tensile strength and elongation at break Improved the antioxidant activity Antimicrobial activity against *E. coli* and *S. aureus*	TS(MPa): 2.53 ± 0.18, 19.24 ± 0.61 (respectively, for zein, zein–8% catechin-loaded β-cyclodextrin metal) EB (%): 1.65 ± 0.04, 4.51 ± 0.14 (respectively, for zein, zein–8% catechin-loaded β-cyclodextrin metal)	-	Potential active food packaging material	[177]
Collagen					
Collagen–chitosan–lemon essential oil	Improved tensile strength, elongation at break and low oxygen permeability	TS (MPa): 30.97 ± 5.26, 20.73 ± 3.88 (respectively, for collagen, collagen–chitosan–lemon essential oil 40%) EB (%): 65.41 ± 10.28, 84.57 ± 11.12 (respectively, for collagen, collagen–chitosan–lemon essential oil 40%)	-	Delay deterioration of pork at 4 °C for 21 days by preventing lipid oxidation and microbial proliferation	[60]
Collagen–alginate-SiO_2_	Reduction in moisture content, water vapor transmission rate, and water vapor permeability	-	-	Potential active food packaging material	[169]

PLA shows incredible properties for films for food packaging applications such as mechanical strength, light transmission, transparency, rigidity, low cost, high stiffness, resilience, biodegradability, excellent barrier properties, and biocompatibility. However, it is brittle, has low melt strength, and has low thermal stability [9,62,84,98].

In recent years, many different types of nanofillers have been incorporated into the PLA matrix. Kostic et al. [62] incorporated alginate microbeads containing AgNPs into a PLA matrix to form a nanocomposite film by using the solvent casting method. Here, the PLA matrix acted as a diffusion barrier by lowering the Ag migration levels within the allowed limit of 0.05 mg kg^−1^ after 10 days. Further, the films had inhibitory effects against *S. aureus*. While the incorporation of modified carbon nanostructures in the PLA matrix increases the thermal and mechanical properties of the film [84]. On the other hand, the studies of Andrade-Del Olmo et al. [178] focused on forming a layer-by-layer bio-nanocomposite film of PLA-ZnONPs–chitosan-β-cyclodextrins. Here, PLA was blended internally with ZnONPs, and it was superficially modified by the deposition of chitosan and cyclodextrins. The microbial properties of treated surfaces were improved as a result of increased surface hydrophilicity. The multilayers appear to be acceptable substrates for carvacrol loading and release, with maximum release occurring during the first 14 days of exposure.

Further, the incorporation of active agents’ thymol, kesum, and curry into a PLA to form an active film through the solvent casting method was carried out in [98]. The films showed antimicrobial activity against *S. aureus*. All active-agent-loaded PLA films successfully kept chicken flesh in good condition during storage for up to 15 days. Yang et al. [67] developed a packaging film by grafting star-like lignin microparticles onto PLA via the ring open polymerization of l-lactide, which began with the hydroxyl groups on the lignin microparticle surface. With the addition of lignin microparticles, elongation at break increased up to 236%, and there was excellent UV resistance behavior, antioxidant activity, and low migration level, making it a suitable packaging material.

#### 6.3.2. Poly (Butylene Adipate Terephthalate) (PBAT)

Poly (butylene adipate terephthalate) (PBAT) is an aliphatic-aromatic copolyester obtained from the poly-condensation of butanediol, adipic acid, and terephthalic acid [95]. It has been used for applications in agricultural, food packaging, and biomedical areas. The major commercially available PBAT biopolymers are manufactured in BASF (ECOFLEX^®^, Ludwigshafen, Germany), KINGFA (ECOPOND^®^, Guangzhou, China), NOVAMONT (Origo-Bi^®^, Novara, Italy), TUNHE (Beijing, China), XINFU (Beijing, China), and JINHUI (ECOWORD^®^, Lvliang, China). Shopping bags have been developed using Starch-PBAT blends by KINGFA, which is widely used in Chinese supermarkets [80].

PBAT has good mechanical and biodegradable properties. However, it has poor photostability, which leads to a decline in its mechanical performance during the application. Furthermore, it has high melt viscosity, low crystallization rate, low tensile strength, and high production cost. Therefore, it is usually used in an application with blended polymers, nanofillers, or other natural compounds [9,66,179]. Thus, blended products of starch-PBAT and PLA-PBAT have been developed by KINGFA, China, to improve the mechanical properties and production cost.

Recent studies have been performed with PBAT blended polymers. Sharma et al. [64] developed a PLA-PBAT–ferulic acid, and this film was incorporated with ferulic acid by using the solvent casting method. The thickness of the film was raised by 1.5–10%, and tensile strength increased to 10.78 MPa from 5.42 MPa (control film) when ferulic acid was added to the film. The film also showed antimicrobial activity against *Listeria monocytogenes* and *E. coli*.

**Figure 8:**
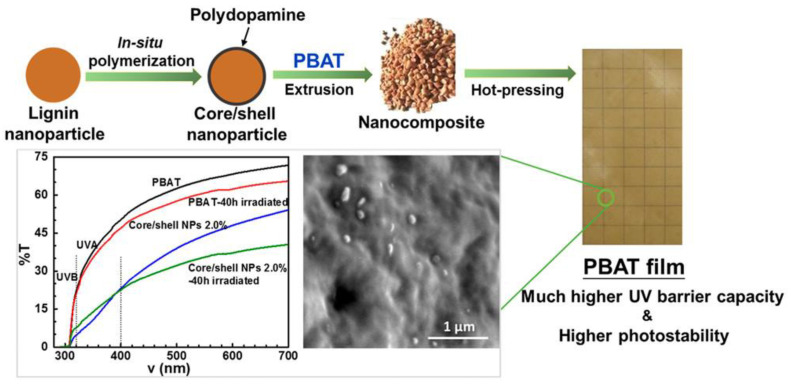
Development of Lignin nanoparticle and PBAT-based food packaging material. Reprinted/adapted with permission from Ref. [35]. 2019, Elsevier.

Further studies have been performed by incorporating lignin or melanin or lignin–melanin core-shell into a PBAT matrix [35] as shown in Figure 8. At 0.5 to 5 wt% NP concentrations, all of these films had outstanding UV-blocking capacity, blocking nearly all UV-A and more than 80% of UV-B light while maintaining reasonable optical transparency.

#### 6.3.3. Polycaprolactone (PCL)

Polycaprolactone (PCL) is a biodegradable polyester with a low melting point of 60 °C and a glass transition temperature of −60 °C. It is prepared by the ring-opening polymerization of ɛ-caprolactone. It has poor mechanical and thermal properties, high solubility, and an excellent ability to form blends. Lukic, Vulic, and Ivanovic, [180] developed a blend packaging material PCL-PLA–thymol–carvacrol by using the solvent casting method. The thymol and carvacrol were loaded into the PCL-PLA mixture by utilizing supercritical CO_2_ at 40 °C and 10 MPa for 5 h. The PCL-PLA film loaded with the thymol and carvacrol mixture had the highest total polyphenol content (128.05 mg GAE/g film) and antioxidant activity (7590.0 μmolTrolox equivalent/g film), acceptable physical properties, and the lowest release rate of 44.51 mg/L released after 6 weeks.

#### 6.3.4. Polybutylene Succinate (PBS)

Polybutylene succinate (PBS) is a thermoplastic biodegradable aliphatic polyester formed by polycondensation. It has properties similar to polypropylene with high crystallinity and good thermal and mechanical properties. PBS has been used as an additive with other biopolymers such as PLA [67]. A blended film of PBS-PLA was developed with the incorporation of carvacrol and thymol by the extrusion casting method [180]. The inclusion of active compounds increased the ductility and flexibility of PLA-PBSA-based active films. PLA-PBSA films with carvacrol or thymol had a high release of the active compound and high antioxidant effectiveness. The spoilage and deterioration of salmon slices were minimized, resulting in a 3–4-day extension of the shelf-life during cold storage.

#### 6.3.5. Polyhydroxyalkanoate (PHAs)

Polyhydroxyalkanoate (PHAs) is a biodegradable, intracellular, and biocompatible family of bacterial polyesters, which are produced by bacterial fermentation of sugar and lipids. They have similar mechanical and thermal properties to synthetic polypropylene. The merits of PHA include good tensile strength, printability, flavor and odor, barrier properties, and temperature stability. The application of PHA is limited due to its high cost. Different polymers of PHAs have been produced by different substrates; Poly-3-(hydroxybutyrate-co-3-hydroxyvalerate) (PHBV) is produced from starch, vinnase, ethanol stillage, and cheese whey, while Poly-3-(hydroxybutyrate) (PHB) is produced from molasses wastewater and date syrup. Similarly, poly-β-hydroxyvalerate (PHV) is produced from molasses wastewater. PHA is used for industrial applications such as in the pharmaceutical, medical products, cosmetics, agriculture, aerospace, and food packaging industries. During industrial applications, PHAs can be used as raw materials or as blends with other polymers such as PLA, PBS, and PCL [181]. PHA is used as a biodegradable packaging application in bottles, containers, sheets, films, laminates, fibers, and coatings manufacturing. Metabolix (US) produces Metabolix PHA (blend of PHB and poly 3-hydroxyoctanoate) for food packaging and additive application [170,182].

**Table 4:** Applications of aliphatic polyester biopolymers in food packaging.

Packaging Material	Characteristics of Food Packaging System	Mechanical Properties	Thermal Properties	Application	Reference
Poly lactic acid (PLA)					
PLA-cellulose nanocrystals–green tea extract	Improved barrier properties, antioxidant activity, and effectiveness in retarding lipid oxidation Reduced oxygen transmission ratio and water vapor permeability	TS(MPa): 39.8 ± 5.8, 36.3 ± 3.5 (respectively, for PLA, PLA-2% cellulose nanocrystals–green tea extract) EB (%): 2.7 ± 0.4, 2.3 ± 0.1 (respectively, for PLA, PLA-2% cellulose nanocrystals–green tea extract)	Tg (°C): 63.2, 59.6 (respectively, for PLA, PLA-2% cellulose nanocrystals–green tea extract)	Extended the shelf-life of salami slices exhibiting an oxidation reduction	[65]
PLA—alginate microbeads containing silver NPs	Improved thermal properties and Young’s modulus and reduced elongation at break Antimicrobial activity against *S. aureus*	TS(MPa): 15.5 ± 1.5, 14.0 ± 1.1 (respectively, for PLA, PLA composite) EB (%): 477 ± 26, 77 ± 23 (respectively, for PLA, PLA composite)	-	Potential active food packaging material	[94]
PLA—carbon NPs	Improved the thermal and mechanical resistance	-	Tg (°C): 280, 215 (respectively, for PLA, PLA—0.09% carbon nanotubes)	Potential active food packaging material	[183]
PLA—thymol–kesum–curry	Improved thermal stability and water vapor barrier properties Antimicrobial activity against *S. aureus* and no antimicrobial activity against *E. coli*	-	Initial decomposition temperature (°C): 352.9, 342.7 (respectively, for PLA, PLA—thymol–kesum–curry)	Increased shelf-life of chicken up to 15 days. Active packaging material suitable for meats, fruits, and vegetables products	[98]
PLA—lignin micro particles	Improved elongation at break, UV resistance, and antioxidant activity	-	Tg (°C): 62.1 ± 0.3, 64.8 ± 0.4 (respectively, for PLA, PLA—ethylene−vinyl acetate−glycidyl methacrylate)	Potential active food packaging material	[184]
PLA—ZnO NPs	Enhanced thermal stability Antimicrobial activity against *S. aureus* and *E. coli*.	-	Tg (°C): The pure PLA film has shown Tg around 60 °C and Tm around 156 °C	Potential active food packaging material	[185]
PLA—fenugreek essential oil-curcumin	Improved UV barrier properties, surface color, tensile strength, flexibility, thickness, and water contact angle Enhanced antibacterial and antioxidant properties	TS(MPa): 30.27 ± 1.0, 36.79 ± 0.88 (respectively, for PLA, PLA—fenugreek essential oil–curcumin) EB (%): 16.68 ± 1.68, 53.08 ± 5.12 (respectively, for PLA, PLA—fenugreek essential oil–curcumin)	Tg (°C): 58.67, 63.02 (respectively, for PLA, PLA—fenugreek essential oil–curcumin)	Potential active food packaging material	[186]
PLA—PBAT-tannic acid–gallic acid	Enhance UV blocking properties and surface hydrophobicity Antimicrobial activity against *E. coli* and *L. monocytogenes*	TS(MPa): 4.80 ± 0.06, 8.63 ± 0.3, 7.01 ± 0.95 (respectively, for PLA, PLA-PBAT-10% tannic acid, PLA, PLA-PBAT—10% gallic acid) EB (%): 21.94 ± 11.42, 23.52 ± 9.18, 22.09 ± 18.64 (respectively, for PLA, PLA-PBAT—10% tannic acid, PLA, PLA-PBAT—10% gallic acid)	The first weight loss at around 30 to 70 °C	Potential active food packaging material	[187]
Poly(butylene adipate terephthalate) (PBAT)					
PBAT-PLA—ferulic acid	Improved tensile strength and UV light barrier properties Antibacterial activity against *L. monocytogenes* and *E. coli*	TS(MPa): 5.42 ± 0.03, 10.78 ± 0.83 (respectively, for PBAT-PLA, PBAT-PLA—ferulic acid) EB (%): 21.93 ± 17.42, 22.13 ± 21.34 (respectively, for PBAT-PLA, PBAT-PLA—ferulic acid)	The first weight loss stage was around 60 to 80 °C	Potential active food packaging material	[64]
PBAT-lignin—melanin NPs	Improved UV barrier capability, photostability, tensile properties, and thermal stability		One-step degradation process with an initial decomposition temperature of 369 °C major weight loss occurring around 400 °C	Potential active food packaging material where high UV resistance is required	[35]
PBAT-PLA—nano-polyhedral oligomeric silsesquioxane	Improved mechanical properties, water vapor, CO_2_ and O_2_ permeability	-	-	Potential active food packaging material	[9]
PBAT—glycerol–zeolite–citric acid–cassava starch	Improved mechanical properties and Water vapor permeability	TS(MPa): 2.44 ± 0.23, 2.44 ± 0.24 (respectively, for control films, zeolites) EB (%): 74.84 ± 23.74, 97.74 ± 19.99 (respectively, for control films, zeolites)		Preserved the color and vitamin C content broccoli florets for 7 days. Senescence indicator of labels were able to detect CO_2_ in packages	[188]
PBAT-PLA—carvacrol	Reduced permeation of vapor and oxygen Delayed fungal growth and sporulation of *Penicillium* sp. and *Rhizopus* sp. Increased shelf-life of packaged bread and butter cake by 2.0–2.3 times	TS(MPa): 26.8 ± 3.9, 16.4 ± 1.4 (respectively, for PBAT 70-PLA 30, PBAT 70-PLA 30—5% carvacrol) EB (%): 267.3 ± 37.3 (respectively, for PBAT 70-PLA 30, PBAT 70-PLA 30—5% carvacrol)	Weight loss at degradation temperatures of 100, 310 and 350 °C	Potential active food packaging material	[189]
PBAT—zinc oxide–graphene oxide	Improved the mechanical and thermal properties Antibacterial activity against *E.coli* (81.32%) and *S. aureus* (82.44%)	TS(MPa): 7.65 ± 0.55, 27.43 ± 0.83 (respectively, for PBAT, PBAT—zinc oxide–graphene oxide) EB (%): 121.96 ± 6.35, 304.38 ± 14.84 (respectively, for PBAT, PBAT—zinc oxide–graphene oxide)	Final thermal decomposition at about 650 °C	Potential active food packaging material	[190]
PBAT—SiO_2_ NP-grape seed essential oil	Improved antimicrobial activities, film flexibility, and optical and heat resistance properties	TS(MPa): 35, 43 (respectively, for PBAT, PBAT-GEO-SiO_2_NP (87:10:3)) EB (%): 590, 595 (respectively, for PBAT, PBAT-GEO-SiO_2_NP (87:10:3))	Initial weight loss at temperatures of 70–90 °C. Second thermal decomposition at 320–411 °C	Potential active food packaging material	[179]
Poly caprolactone (PCL)					
PCL-PLA—thymol–carvacrol	High total polyphenol content, increased antioxidant activity, good storage stability, acceptable physical properties, and low release rate	TS(MPa): 29.6 ± 1.47, 6.42 ± 0.6783 (respectively, for PCL–PLA, PCL-PLA—thymol–carvacrolzinc oxide–graphene oxide) EB (%): 603.4 ± 48.7, 10.68 ± 2.30 (respectively, for PCL –PLA, PCL-PLA-thymol–carvacrolzinc oxide–graphene oxide)	-	Potential active food packaging material	[180]
PCL-α-tocopherol	High antioxidant activity Reduction of microbial growth on cheese	-	-	Potential active food packaging material	[191]
PCL-poly(propylene carbonate)	Improved gas barrier property and water vapor permeability Extended the shelf-life of button mushroom up to 17 days of storage.	TS(MPa): 9.6 ± 1.0, 19.9 ± 0.9 (respectively, for PCL-40, PCL-60) EB (%): 371 ± 43.9, 465 ± 36.9 (respectively, for PCL-40, PCL-60)	-	Potential active food packaging material	[192]
Poly (butylene succinate adipate) (PBSA)					
PBSA-PLA	Improved mechanical, antibacterial, and antioxidant properties	TS(MPa): 48.61 ± 1.22, 36.68 ± 1.74 (respectively, for 90 wt% PLA +10 wt% PBSA, 82.8 wt% PLA +9.2 wt% PBSA +8 wt% Thymol) EB (%): 55.70 ± 3.56, 353.80 ± 24.80b (respectively, for 90 wt% PLA +10 wt% PBSA, 82.8 wt% PLA +9.2 wt% PBSA +8 wt% Thymol)	Endothermic peak of melting at 149 °C	Extended the shelf-life of salmon slices by 3–4 days during cold storage. Active packaging material suitable for fishery products	[67]
PBSA-poly(3-hydroxybutyrate-co-3-hydroxyvalerate)	Improved melt viscosity and accelerated crystallization kinetics	TS(MPa): 2153, 1297 (respectively, for PHBV/PBSA 100/0, PHBV/PBSA 70/30) EB (%): 0.98 ± 0.1, 134.8 ± 48 (respectively, for PHBV/PBSA 100/0, PHBV/PBSA 70/30)	Tm (°C): 88 ± 3, 86 ± 2, (respectively, for PHBV/PBSA 0/100, PHBV/PBSA 70/30) Tg (°C): −45.9 ± 1.6, −48.6 ± 2.3 (respectively, for PHBV/PBSA 0/100, PHBV/PBSA 70/30)	Potential active food packaging material	[193]
Polyhydroxyalkanoate (PHAs)					
PHB-graphene nanoplatelets	Improved thermal stability, barrier properties, and tensile strength Decreased oxygen and water vapor permeability Statistically insignificant cytotoxic effect	TS (MPa): 4.5, 12.2 (respectively, for PHB, PHB-1.3 wt% graphene nanoplatelets)	Tmax: 279.4 °C, 284.1 °C (respectively, for PHB, PHB-1.3 wt% graphene nanoplatelets)	Active packaging material suitable for moisture and oxygen-sensitive food items (potato chips and milk product)	[190]
PHB-polycaprolactone-organo-clays (Cloisite^®^ 30 B and 10A)	Improved barrier properties and degradation temperature Antimicrobial activity against *Lactobacillus plantarum*	TS(MPa): 6.29 ± 1.42, 7.06 ± 1.96 (respectively, for PHB-PLA, PHB-PLA-Cloisite^®^ 30 B) EB (%): 3.03 ± 1.71, 0.72 ± 0.19 (respectively, for PHB-PLA, PHB-PLA-Cloisite^®^ 30 B)		Increased shelf-life of sliced ham. Active packaging material suitable for processed meat packaging	[69]
PHBV-PHB-eugenol	Improved hydrophobicity, mechanical, and thermal barrier properties. Strong adhesion and high electro spinnability Antimicrobial activity against *S. aureus* and *E. coli*	TS(MPa): 1491 ± 207, 1446 ± 190 (respectively, for active multilayer with cellulose nanocrystal, active multilayer with cellulose nanocrystal) EB (%): 59.1 ± 56, 51.6 ± 45 (respectively, for active multilayer with cellulose nanocrystal, active multilayer with cellulose nanocrystal)	-	Potential multilayer antimicrobial active food packaging material	[70]
PHBV-PHB–cellulose nanofibrils-lignocellulose nanofibrils	Improved water contact resistance, mechanical and water vapor and oxygen barrier properties Slightly lower aroma barrier properties	TS(MPa): 4504.2 ± 105, 2991.4 ± 184 (respectively, for cellulose nanofibrils, lignocellulose nanofibrils) EB (%): 18.1 ± 2.2, 13.7 ± 0.5 (respectively, for cellulose nanofibrils, lignocellulose nanofibrils)	-	Potential active food packaging material	[194]
PHBV-thermoplastic starch	Improved oxygen barrier properties, reduce water uptake Oxygen barrier properties are slightly compromised	-	-	Build on current knowledge on multilayered TPS-PHBV film for food packaging applications	[195]
PHA-cellulose nanocrystals	Good interlayer adhesion and contact transparency Enhanced mechanical properties	TS(MPa): 24.5 ± 0.6, 39.0 ± 1.9 (respectively, for poly(3-hydroxybutyrate-co-3-hydroxyvalerate) (PHBV) containing 8 mol.%, 2 mol.% EB (%): 2.6 ± 0.2, 1.4 ± 0.1 (respectively, for poly(3-hydroxybutyrate-co-3-hydroxyvalerate) (PHBV) containing 8 mol.%, 2 mol.%	-	Potential active food packaging material	[182]

Polyhydroxybutyrate (PHB) is the most abundant PHAs that is a biodegradable lipid-like polymer synthesized by different bacteria that has a rigid structure. It is suitable as a food packaging material since it is renewable, biocompatible, and has low oxygen and water permeability and increased barrier properties. A study by Rech et al. [193] combined PHB with essential oils (cinnamon, melaleuca, and citronella) to form an edible film for food packaging by using the solution casting method. Essential oils increased both the crystallinity degree and the thermal stability of PHB films. Furthermore, by lowering the melting temperature from 155.7 °C to 143.7 °C (melaleuca-cinnamon) and increasing film flexibility by the reduction in the elastic modulus from 1030 MPa to 286 MPa (melaleuca-cinnamon) of the polymer, these oils created a plasticizing effect. Additional studies on PHB incorporated with NPs were performed by PHB-graphene nanoplatelets [69] and PHB-polycaprolactone (PCL)-organo-clays [70]. Manikandan et al. [69] formed a PHB nanocomposite film by incorporating different concentrations of graphene nanoplatelets (0–1.3 wt%) via the solution casting method. The incorporation of graphene nanoplatelets into PHB increased the melting point by 10 °C, thermal stability (by 10 °C), tensile strength by 2 times, and reduced oxygen and water vapor permeability by 3 and 2 times, respectively. Further, there was a four-fold increase in the shelf-life of potato chips and milk products. Correa et al. [70]] incorporated organo-clays (Cloisite^®^ 30 B and 10A) into a matrix of PHB/PCL by melt intercalation, and the nanocomposite films were formed by compression molding. Although organo-clays have antimicrobial activity against *Lactiplantibacillus plantarum*, their incorporation in the polymer blend did not result in antimicrobial films. However, the nisin-activated PHB-PCL film showed antimicrobial activity against *Lactiplantibacillus plantarum* by prolonging the shelf-life of sliced ham. Poly(3-hydroxybutyrate-co-3-hydroxyvalerate) (PHBV) has higher processability and physical properties than PHB. PHBV also has high flexibility, toughness, and low melting point. Recently, Figueroa-Lopez et al. [170] developed a multilayer PHBV-PHB-eugenol active food packaging material by incorporating different concentrations of eugenol into the PHBV ultra-thin fibers by electrospinning. Then, this PHBV monolayer was interlaid between PHB sheets by annealing at 160 °C for 10 s to form a multilayer active packaging material. This film showed high hydrophobicity of 75.53 and improved mechanical (tensile strength improved from 1252 MPa to 2884 MPa with the addition of 2.5 wt.% of eugenol to PHBV), thermal, barrier, and antimicrobial (against *S. aureus* and *E. coli*) properties. Further studies were performed on developing PHBV-PHB–cellulose nanofibrils–lignocellulose nanofibrils containing mono- and multilayer films by using the electrospinning coating technique [170]. The multilayer film with PHBV-PHB–cellulose nanofibrils–lignocellulose nanofibrils reduced oxygen permeance by 35% when compared to the control film, thus having enhanced gas barrier performance.

## 7. SWOT Analysis of Biodegradable Polymers in the Food Packaging Industry

### 7.1. Strengths

Biopolymers are eco-friendly, biodegradable, nontoxic, renewable, and biocompatible alternatives to synthetic packaging materials. These are easily recycled, avoiding the environmental pollution caused by synthetic polymers while addressing the important question of environmental pollution. Thus, it is environmentally friendly and possesses a much lower risk than synthetic products. Biopolymers are naturally occurring in animals, plants, and microorganisms and are thus highly abundant. The extraction process and the synthesis depend on the different biopolymers.

They have great film-forming ability and different strengths specific to each biopolymer as discussed above. Biopolymers can create high-performance packaging materials together with other biopolymers or reinforcement agents, and they are lightweight. Biopolymers work as matrices to incorporate nanofillers, natural compounds, antimicrobial agents, antioxidants, vitamins, minerals, and nutrients to make them more suitable active packaging materials.

### 7.2. Weaknesses

Even though biopolymers are environmentally friendly, their main weaknesses are low mechanical and barrier properties, rapid degradation rate, high hydrophilic capacity, and high sensitivity to moisture. In addition to the poor material performance, they also have weak mechanical and chemical structures, making many of the biopolymers not suitable for food packaging directly. Further, when compared to synthetic polymers, they are difficult to process and relatively expensive. Most of the biopolymers are naturally hydrophilic and deteriorate when exposed to moisture. The above-mentioned characteristics of the biopolymers make them unsuitable as packaging materials since it makes them unsuitable to maintain the shelf-life and quality of food products.

### 7.3. Opportunities

There are a wide variety of opportunities for using biopolymers in food packaging, including active and smart food packaging materials. Biopolymers are the base compound for most of the packaging material combinations along with nanomaterials or other active compounds. The addition of the reinforcement agents into the biopolymer matrix results in the improvement of essential properties needed in packaging material, such as barrier, mechanical, thermal, antioxidant, and antimicrobial properties. Most of the food packaging materials developed are still in the research stage, and there is an opportunity for the upscale and global production of these materials to use as an alternative to synthetic polymers. Biopolymers and bioplastic industrial production possess the opportunity to reduce global environmental pollution and aid the circular economy as mentioned by the European Union.

### 7.4. Threats

While environmental concerns regarding plastic packaging are well-documented, the assessment of biopolymers’ environmental impact is more nuanced. Biopolymers have the potential to be more environmentally friendly, but their specific advantages and disadvantages hinge on a number of variables, such as their origin, production processes, waste management systems, and end-of-life considerations. To make informed decisions about a material’s environmental impact, it is essential to conduct a thorough analysis of the material’s life cycle. Most of the biopolymers in their pure form do not deliver a threat to society or the environment. However, these biopolymers are combined with nanofillers or other active agents to improve the qualities of the packaging materials. These agents pose a threat of migration into a food product and gradually migrate into the human body. This may cause a threat to human health if the agent is cytotoxic. Further, during the biodegradation process, the active agents are migrated into soil/water, which may affect environmental conditions, leading to pollution. The migration of chemical compounds is not unique to biopolymers; it can also occur with other packaging materials. It is essential to assess and manage the potential migration of compounds from any packaging material, including biopolymers, to ensure food safety and regulatory compliance. Ongoing research and development is aimed at enhancing the safety and efficacy of biopolymers for food packaging applications. This includes the development of novel materials, the optimization of processing techniques, and extensive testing to ensure their suitability for food contact and to minimize the migration of potentially hazardous substances. In addition, the microorganisms used to produce biopolymers may be hazardous and may result in environmental pollution.

## 8. Future Trends and Conclusions

Over the years, synthetic packaging materials have been the primary source of food packaging. However, the use of synthetic polymers presents challenges and limitations, mainly due to environmental pollution issues caused by plastics. Consequently, the trend of using biopolymers in food packaging has significantly increased in recent years. Biopolymers/bioplastics such as thermoplastic starch, PLA, cellulose, and PBAT are already used in industrial production for food packaging applications. Their characteristics, such as biodegradability, eco-friendliness, renewability, nontoxicity, and lightweight properties, make them suitable for food packaging applications. However, the applications of biopolymers in their pure form are limited due to their low mechanical, barrier, and thermal properties. Furthermore, they are less cost-effective compared to synthetic polymers. The negative characteristics of biopolymers can be overcome by adding reinforcement agents such as nanofillers and active agents. These reinforcing agents enhance the properties of the packaging materials, making them suitable for active and intelligent packaging materials by extending their shelf-life and enhancing the quality of packaged food products. In recent years, researchers have focused on conducting studies on the combinations of biopolymers, reinforcement agents, and their applications. However, there are limited studies carried out using some biopolymers; for instance, the natural UV barrier property of lignin has been studied in limited research. Moreover, when compared to starch-based biopolymers, studies on protein biopolymers and some aliphatic biopolymers (PBS, PCL) are limited. The food packaging applications of the produced biopolymers have only become an interest to scientists in recent years, and there is still considerable room for improvement in research. Furthermore, only a few studies have been performed to evaluate the cytotoxicity effect of the produced packaging material and the biodegradation ability of the materials. These two aspects of the study are crucial to avoid global issues in the future. Additional studies are warranted to bring biopolymer-based food packaging to a global level and use it as an alternative to plastic packaging.

## Figures and Tables

**Table 1:** Advantages and disadvantages of different biopolymers in food packaging.

Biopolymer	Positive Characteristics	Negative Characteristics	References
Starch-based biopolymers			
Starch	Biodegradable Renewable Nontoxic Low cost Abundance Transparent colorless, flavorless, tasteless Good lipids, oxygen, UV barrier properties Great film-forming ability Low water vapor permeability	Limited process ability Poor water resistance Low mechanical properties Hydrophilic Low thermal properties Brittleness	[1,6,7,8,9]
Chitosan	Biodegradable Renewable Nontoxic Increased absorption properties High antimicrobial activity High biocompatibility Low production cost Good gas, aroma, UV, oil barrier properties Wettability Antioxidant properties water-insoluble Good film-forming ability Good optical properties Transparent Flexible	Low mechanical properties High hydrophobicity Low water vapor barrier properties Brittleness Low elasticity	[10,11,12]
Carrageenan	Biodegradable Renewable Nontoxic Good gas, moisture barrier properties Thermal stability Antibacterial properties Excellent film-forming ability Transparent	Poor mechanical properties Low water vapor barrier properties Water resistance properties	[13,14,15,16,17]
Cellulose	Biodegradable Renewable Nontoxic Low energy consumption High surface area Good oxygen, hydrocarbon barrier properties High mechanical strength High water vapor permeability Low cost Low density High specificity Biocompatibility Odorless, tasteless Chemical stability	Low mechanical strength Opacity Enhanced color value Hydrophilic nature Poor water vapor barrier properties	[18,19,20,21]
Agar	Biodegradable Renewable Nontoxic Good film-forming ability Stability in different environment conditions Transparent	Poor water vapor barrier properties Poor mechanical properties Brittleness Poor thermal stability Strong hydrophilic characteristic	[3,4,22,23,24]
Pectins	Biodegradable Renewable Nontoxic Good oil, aroma, gas barrier properties High mechanical properties Good rheological properties Cost effective Good film-forming capacity	Ineffective against moisture transfer Poor mechanical properties Brittleness Poor thermal stability High water solubility Lack of antimicrobial properties	[25,26,27,28]
Alginate	Biodegradable Renewable Nontoxic Control swelling properties Low cost Biocompatibility Good mechanical properties Chemical stability Good water barrier properties Good mechanical properties Stiffness Maintaining the flavor Retarding fat oxidation	Brittleness Poor moisture barriers Poor water resistance High water vapor permeability High hydrophilicity	[29,30,31,32,33]
Gums	Biodegradable Renewable Nontoxic Control viscosity Biocompatibility Low cytotoxicity	High cost of production Low rheological properties Low mechanical properties Low barrier properties	[34,35,36]
Lignin	Biodegradable Renewable Nontoxic Natural broad UV blocker Antioxidant properties	Low mechanical properties Low barrier properties	[4,34,37,38]
Pullulan	Biodegradable Renewable Nontoxic Odorless, tasteless, colorless Flexible Transparent Thermal stability Good oil, oxygen barrier properties Biocompatibility Heat-sealable water permeable	Low mechanical properties Brittleness Low water resistance Moisture sensitivity	[20,39,40]
Curdlan	Biodegradable Renewable Nontoxic Colorless, odorless High absorption Water insoluble Thermal stability	Poor mechanical properties	[41,42]
Protein-based biopolymers			
Gelatin	Biodegradable Renewable Nontoxic Low cost Abundant Excellent film-forming ability Biocompatible Flexible Transparent Excellent water, UV, aroma oxygen barrier properties Low water vapor permeability	Poor swelling properties Low tensile strength Opacity High roughness Poor mechanical properties Poor processability	[24,43,44,45]
Soy protein	Biodegradable Renewable Nontoxic Good oxygen, lipid barrier properties Abundance Low cost Biocompatibility Excellent film-forming capacity High water vapor permeability	Low water resistance Low thermoplasticity Brittleness Low mechanical properties Low film gloss Low tensile strength Poor plasticity Low water vapor permeability	[46,47,48,49]
whey proteins	Biodegradable Renewable Nontoxic Tasteless, flavorless Flexible Transparent Soft Elastic water-insoluble Good gas, aromatic, grease barrier, oxygen barrier properties Low cost Nutritional value Excellent film-forming ability	Weak resistance to moisture Low mechanical properties	[50,51,52,53]
zein	Biodegradable Renewable Nontoxic Good oxygen/gas barrier properties High thermal resistance High tensile strength Hydrophobic properties High antimicrobial potential Good antioxidant properties Form adhesive film High toughness Low water vapor permeability	Low flexibility Low mechanical strength Brittleness High relative humidity condition Poor processability Low elongation at break Weak thermal properties Weak mechanical properties Rapid dissolution rate Low gas permeability	[54,55,56,57]
Keratin	Biodegradable Renewable Nontoxic Hydrophobic properties	Poor mechanical properties	[58,59]
Collagen	Biodegradable Renewable Nontoxic Excellent film formation ability Biocompatibility Antioxidant properties Good moisture, oxygen barrier properties Ensure structural integrity	Poor mechanical strength High water vapor permeability	[60]
Aliphatic polyester-based biopolymers			
Poly lactic acid (PLA)	Biodegradable Renewable Nontoxic Good flavor, odor Antimicrobial properties Transparent Good oil, oxygen barrier properties Good mechanical strength Light transmission Rigidity Low cost High stiffness Flexibility Biocompatibility	Brittleness Low mechanical properties Low thermal stability Low melt strength	[61,62,63,64,65]
poly(butylene adipate terephthalate) (PBAT)	Biodegradable Renewable Nontoxic Flexible Good oxygen barrier Chemical inactivity High viscosity Good mechanical properties Low water vapor permeability	Poor resistance Poor impact resistance High cost of production Low thermal properties Low antimicrobial activity Poor photostability Low mechanical performance Low crystallization rate	[9,63,66]
Poly caprolactone (PCL)	Biodegradable Renewable Nontoxic Excellent ability to form blends	Poor mechanical properties Poor thermal properties High solubility	[57]
polybutylene succinate (PBS)	Biodegradable Renewable Nontoxic High crystallinity Good thermal properties Good mechanical properties	Poor barrier properties	[67]
Polyhydroxyalkanoate (PHAs)	Biodegradable Renewable Nontoxic Good tensile strength Flavor, odor Good oxygen, water barrier properties Temperature stability Biocompatible	High cost Low thermal properties Low mechanical properties	[68,69,70]

## Data Availability

The data used to support the findings of this study can be made available by the corresponding author upon request.
